# Effect of particle size and composition on local magnetic hyperthermia of chitosan-Mg1−xCoxFe2O4 nanohybrid

**DOI:** 10.3389/fchem.2024.1347423

**Published:** 2024-03-07

**Authors:** M. Aminul Islam, Ishtiaque M. Syed, M. Al Mamun, S. Manjura Hoque

**Affiliations:** ^1^ Materials Science Division, Atomic Energy Centre Dhaka, Bangladesh Atomic Energy Commission, Dhaka, Bangladesh; ^2^ Department of Physics, University of Dhaka, Dhaka, Bangladesh; ^3^ Department of Physics, Magura Govt. Mahila College, Magura, Bangladesh

**Keywords:** hyperthermia, specific loss power, magnesium ferrite, cobalt ferrite, Raman spectra

## Abstract

In this study, Mg_1*−x*_Co_*x*_Fe_2_O_4_ (0*≤*x *≤ 1* with ∆x = 0.1) or MCFO nanoparticles were synthesized using a chemical co-precipitation method and annealed at 200, 400, 600, and 800°C respectively to investigate the structural properties of the materials by X-ray diffractometer (XRD), transmission electron microscopy (TEM), and Fourier-transform infrared spectroscopy (FTIR). Controlled annealing increased particle size for each value of x. The aim was to investigate how specific loss power (SLP) and maximum temperature (T_*max*_) during local magnetic hyperthermia were affected by structural alterations associated with particle size and composition. The lattice parameter, X-ray density, ionic radius, hopping length, bond length, cation-cation distance, and cation-anion distance increase with an increase in Co^2+^ content. Raman and FTIR spectroscopy reveal changes in cation distribution with Co^2+^ content and particle size. Magnetic properties measured by the physical property measurement system (PPMS) showed saturation magnetization (M_s_), coercivity (H_c_), remanent magnetization (M_r_/M_s_), and anisotropy constant (K_1_) of the Mg_1*−x*_Co_*x*_Fe_2_O_4_ nanoparticles increase with Co^2+^ content and particle size. When exposed to an rf magnetic field, the nanohybrids experienced an increase in both the SLP (specific loss power) and T_max_ (maximum temperature) as the particle size initially increased. However, these values reached their peak at critical particle size and subsequently decreased. This occurs since a modest increase in anisotropy, resulting from the presence of Co^2+^ and larger particle size, facilitates Néel and Brownian relaxation. However, for high anisotropy values and particle size, the Néel and Brownian relaxations are hindered, leading to the emergence of a critical size. The critical size increases as the Co^2+^ content decreases, but it decreases as the Co^2+^ content increases, a consequence of higher anisotropy with the increase in Co^2+^. Additionally, it is noteworthy that the maximum temperature (T_max_) rises as the concentration of nanohybrids grows, but the specific loss power (SLP) decreases. An increased concentration of chitosan-MCFO nanohybrids inhibits both the Néel and Brownian relaxation processes, reducing specific loss power.

## 1 Introduction

Magnetic nanoparticles demonstrate promise for biomedical applications that need smaller particle sizes with superparamagnetic or ferromagnetic behavior. Magnetic nanoparticles are injected intravenously or directly into the malignant cell after appropriate functionalization. Incompatibility is a grave concern for biomedical applications for any physiological implant (Tran and Webster, 2010; Bao et al., 2015; Rajan and Sahu, 2020). Magnesium ferrite is biocompatible because the tolerable limit of magnesium and iron in the human body is higher. According to the Food and Drug Administration of the United States, the reference Daily Intake RDI of Fe and Mg is 18 and 420 mg, respectively. In the case of local magnetic hyperthermia, the temperature range that destroys malignant cells is around 42°C–46°C (Liu et al. 2020). Magnetic nanoparticles with a higher magnetic moment and anisotropy constant increase the efficiency of hyperthermia treatment (Liu et al., 2000; Darwish et al., 2019; Mohapatra et al., 2019). The substitution of magnesium by cobalt in magnesium ferrite and heat treatment can optimize the particle size, magnetic moment, and anisotropy constant, which makes it suitable for hyperthermia application (Nlebedim et al., 2010; Abenojar et al., 2016; Kafrouni and Savadogo, 2016). A higher surface-to-volume ratio of the nanoparticle may adhere them to the walls of the blood vessel, which needs appropriate coating for rolling the particles, transport to a targeted region, and attaining biocompatibility (Fang et al., 2018; Li et al., 2018).

The magnetic properties of ferrite nanoparticles, such as saturation magnetic moment and magnetic anisotropy, are highly structurally sensitive. Therefore, a detailed study of the structure by X-ray diffraction with composition and particle size is worthwhile. Cation distributions of the tetrahedral (A) and octahedral (B) sites play a determining role in controlling the magnetic properties of ferrites (Vijaya and Thyagarajan 2015; Tatarchuk et al. 2017). By substituting non-magnetic magnesium ions with cobalt, one can optimize the structure-property relationship, especially saturation magnetic moment, coercive field, and anisotropy constant, which contribute to the Néel and Brownian relaxation mechanism responsible for particle heating in case of local magnetic hyperthermia treatment (Nemala et al., 2015; Martinez-Boubeta et al., 2013).

In magnetic particle hyperthermia, the particles flow with blood to the targeted region either for the purpose of drug delivery, local magnetic hyperthermia, or *in situ* chemotherapy/hyperthermia or radiotherapy/hyperthermia to kill localized or deeply seeded tumors (Mcbain et al., 2008; Sensenig et al., 2012). In the case of magnetic hyperthermia, magnetic nanoparticles generate heat by the Brownian and Néel relaxation processes when subjected to an alternating magnetic field. The heat is generated by all three mechanisms of the Néel relaxation process, the Brownian relaxation process, and hysteresis loss. However, which mechanism will dominate depends on the particle size and composition of the particles. Consequently, each composition of the nanoparticles would have a critical diameter, which would bear an optimum magnetic moment and anisotropy of the particle (Sensenig et al. 2014; Torres et al., 2019). Therefore, strict control of particle size to optimize magnetization and anisotropy is of utmost importance to tune T_max_ and SLP.

Magnesium is a biocompatible cation that is essential for the human body. For adults, the recommended daily allowances for magnesium are 420 and 320 mg for men and women, respectively. Magnesium is a cofactor for more than 300 enzymes. It plays the most vital role in muscle contraction, neuromuscular conduction, glycemic control, myocardial contraction, bone development, and blood pressure (Alawi et al., 2018). Cobalt is also an essential element for humans. It is present in the human body as cobalamin (vitamin B_12_). Approximately 1 mg/mL of cobalt is present in the adult human body, where 85% of it is in the form of vitamin B_12_. The human daily allowable intake of cobalt is 5–50 *µ*g (Chen and Lee 2023). Though cobalt is more toxic than magnesium, a small amount of magnesium replaced with cobalt would enhance the magnetic properties of Mg_1*−x*_Co_*x*_Fe_2_O_4_, which reduces the required dosage of the magnetic particle in hyperthermia treatment. A small number of nanoparticles as implant alone could reduce the toxicity significantly. Surface coating with chitosan also remarkably reduces the toxicity of the nanoparticle. Considering all these facts, optimization of the parameters of hyperthermia by tuning composition and particle size is crucial, where the combination of cobalt- and magnesium-mixed ferrite with the variation of particle size bears promise for tailoring the structure-property relationship.

In this study, we will synthesize Mg_1*−x*_Co_*x*_Fe_2_O_4_ (where 0 *≤*x *≤ 1* with ∆x = 0.1), i.e., MCFO and chitosan nanohybrids by chemical co-precipitation and vary the core size of the MCFO by heat treatment at the temperatures of 200, 400, 600, and 800°C. We will study the tailoring effect on the efficiency of hyperthermia by estimating the specific loss power, SLP, and maximum temperature, T_max_, for each composition and core particle size.

## 2 Materials and methods

### 2.1 Synthesis technique

In this study, a series of Mg_1*−x*_Co_*x*_Fe_2_O_4_ (where x = 0 to 1.0 with ∆x = 0.1) were synthesized by the wet chemical co-precipitation method using NaOH as the co-precipitating agent. Analytical grade of Mg(NO_3_)_2_ 6H_2_O, CoCl_2_.6H_2_O, and FeCl_3_ were mixed in the required molar ratio under continuous stirring using a magnetic stirrer at a speed of 400 rpm. Then, 8 M of NaOH solution was added dropwise to the solution and the solution was left until the pH was stable. An extra 6 M NaOH was added dropwise to maintain the pH of the solution to a value of 11–13. The mixture was heated to 353 K for 1 h for the completion of the ferritization reaction. We added highly concentrated NaOH to coprecipitate hydroxides to keep the volume of the solvent at a minimum. The following reaction and side reactions took place while adding NaOH:

$$\left(1-x\right)\text{Mg}{\left({\text{NO}}_{3}\right)}_{2}\cdot 6H_{2}O+2\left(1-x\right)\text{NaOH}=>\;\left(1-x\right)\;\text{Mg}{\left(\text{OH}\right)}_{2}\;↓+2\left(1-x\right){\text{NaNO}}_{3}+6\left(1-x\right)H_{2}O$$

$${\text{xCoCl}}_{2}.6H_{2}O+2\text{xNaOH}=>\;\text{xCo}{\left(\text{OH}\right)}_{2}\;↓+2\text{xNaCl}+6{\text{xH}}_{2}O$$

$$2{\text{FeCl}}_{3}+6\text{NaOH}=>\;2\text{Fe}{\left(\text{OH}\right)}_{3}\;↓+6\text{NaCl}$$

$$\left(1-x\right)\;\text{Mg}{\left(\text{OH}\right)}_{2}+x\;\text{Co}{\left(\text{OH}\right)}_{2}+2\text{Fe}{\left(\text{OH}\right)}_{3}=>\;{\text{Mg}}_{1-x}{\text{Co}}_{x}{\text{Fe}}_{2}O_{4}\;↓+4H_{2}O$$

Combining the above four reactions, the key reaction was

$$\left(1-x\right)\text{Mg }{\left({\text{NO}}_{3}\right)}_{2}\cdot 6H_{2}O+{\text{xCoCl}}_{2}.6H_{2}O+8\text{NaOH}=>\;{\text{Mg}}_{1-x}{\text{Co}}_{x}{\text{Fe}}_{2}O_{4}\;↓+2\left(1-x\right)\;{\text{NaNO}}_{3}+2\text{xNaCl}+10H_{2}O$$

The solvent was cooled to room temperature and the ferrite nanoparticles precipitated. The particles were washed 10 times by centrifugation at 13,000 rpm for 20 min to remove extra NaOH. The removal of NaOH was confirmed by the AgNO_3_ test. The particles were then heated at 343 K for 72 h for complete ferritization. The as-dried MCFO nanoparticles were collected from the petri dish, pelletized, and annealed at 200, 400, 600, and 800°C, respectively.

### 2.2 Coating technique

The annealed samples were ground and coated with a 2% (w/v) chitosan solution. The 2% (w/v) chitosan solution was prepared firstly by adding 2 gm chitosan in 70 mL de-ionized water under continuous stirring at 400 rpm for 72 h. During this process, 7 mL acetic acid was added four times to the solution to dissolve chitosan in water. Then the solution was centrifuged two times at 13,000 rpm to ensure that the solution was free from any impurity. We added 20 mg of annealed MCFO nanoparticles to 1 mL 2% chitosan solution, followed by vortexing and sonication several times to get 20 mg/mL chitosan-MCFO nanohybrid colloidal suspension as a stock. Finally, the other concentrations were prepared by diluting from the stock of the colloid.

**FIGURE 1:**
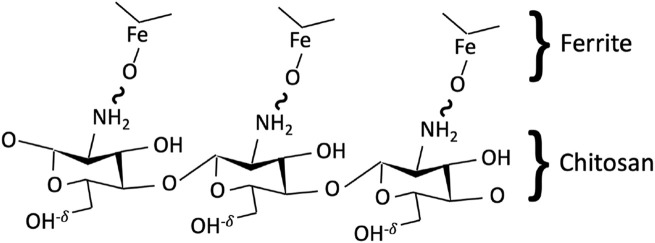
Schematic representation of the bonding between a chitosan polymer chain and Mg_1*−x*_Co_*x*_Fe_2_O_4_ ferrite. The bonding occurs between the oxygen ion of ferrite and hydrogen of the amine group of chitosan, while the hydroxyl group remains free with a slight positive charge, leading to overall particles being positively charged.

Coating/surface functionalization of Mg_1*−x*_Co_*x*_Fe_2_O_4_ ferrite nanoparticles by chitosan occurs because of the bonding between the oxygen of ferrite and the hydrogen of the amine group (-NH_2_) of chitosan. The -OH group remains free, which leads to the net charge of chitosan-Mg_1*−x*_Co_*x*_Fe_2_O_4_ nanohybrid (Rhee et al., 2010) (Figure 1).

### 2.3 Characterization technique

To determine the structural properties of MCFO nanoparticles, X-ray diffraction analysis was performed using a PW 3040 X-ray diffractometer, X’Pert PRO PANalytical model, Philips, Netherlands. A powder sample was subjected to X-ray diffraction (XRD) analysis, with the 2θ angles set between 15 and 70°. The XRD scan was conducted using CuK_α_ radiation, with a voltage of 40 kV and a current of 30 mA. The wavelength of the radiation (λ) was 1.54059 Å. We conducted transmission electron microscopy investigations using a TALOS 200X, manufactured by Thermofisher, United States, operating at a voltage of 200 kV. In order to conduct a TEM investigation, we dispersed the samples in ethanol and dropped them over a carbon-coated Cu grid that is electron-transparent. We dried the drop-cast samples and acquired TEM and HRTEM images and selected area diffraction (SAED) patterns. The Raman spectroscopy measurements were conducted using a CRS+ 500/BX53, MonoVista, S & I Instrument, Germany. A diode laser system operating at a wavelength of 785 nm and with a power of 100 mW was utilized. The laser line was filtered using an edge filter with a width of 60 cm^−1^. Raman spectra were obtained using pellets of samples within the wavelength range of 200–3,500 cm^−1^. The scan step size was set to 0.0167°. The FTIR spectroscopy measurements were acquired using the PerkinElmer machine, United Kingdom, with the attenuated total reflection (ATR) attachment. The powder sample was combined with potassium bromide (KBr) to make pellets and the FTIR spectra was obtained within the range of 350–3,000 cm^−1^. The magnetic properties of all the samples were assessed using the Physical Property Measurement (PPMS) System, Quantum Design, United States, which has a maximum magnetic field strength of 9 T. The time-dependent temperature profiles were obtained using a hyperthermia set-up, namely, the EASY HEAT 5060LI model manufactured by Ambrell in the United States. The hyperthermia set-up comprises a sample coil with eight turns and a diameter of 4 cm. Throughout the hyperthermia experiment, the coil maintained a current of 283 A and a signal frequency of 343 kHz, resulting in a magnetic field of 26 mT at the center of the sample coil. For each test, 600 µL of chitosan-MCFO nanohybrid with varying concentrations was poured in an Eppendorf tube. The tube was then placed at the center of the sample coil and subjected to induction heating for different time intervals under a magnetic field of 26 mT. The temperature was promptly measured with a thermometer right after the power was turned off.

## 3 Results and discussion

### 3.1 Structural characterizations

#### 3.1.1 X-ray diffraction (XRD)

We conducted rigorous studies by X-ray diffraction of the MCFO nanoparticles in the entire range of composition to understand the structure-property relationship, which in turn affected the efficiency of hyperthermia of the nanoparticles. Nanoparticles’ size, shape, and magnetic characteristics affect specific loss power (SLP) and maximum attainable temperature (T_*max*_) of hyperthermia, the ultimate engineering parameters for hyperthermia efficiency. The relaxation phenomena that incur self-heating properties are the Néel and Brownian relaxations and hysteresis loss. Néel relaxation is directly proportional to the volume and anisotropy of the nanoparticles. On the other hand, Brownian motion is directly proportional to the size of the nanoparticles. Nanoparticle size, cation distribution, and magnetic state (ferro/superparamagnetic) affect hysteresis loss. The structure of Co-Mg mixed-spinel ferrites undergoes considerable change throughout the whole spectrum of composition of MCFO due to the nano-magnetism of MgFe_2_O_4_ and CoFe_2_O_4_ at an ultrasmall scale, which again changes with particle size. Hence, acquiring an in-depth understanding of the structure of MCFO nanoparticles at different compositions would contribute to perceiving structure-property relationship, which would affect SLP and T_*max*_ of hyperthermia.

At an annealing temperature of 200°C and when *x* = 0 to 0.3, the XRD pattern exhibits a broad diffuse hump at the 35° position. We delineated in the TEM section that the existence of lattice fringes within the x = 0 to 0.3 range indicates ultra-small particle size. The peaks corresponding to the spinel structure are observable at the annealing temperature of 200°C for the composition, *x ≥* 0.4. Peak shift occurs as x increases due to the difference in the ionic radii of Co^2+^ (0.072 nm) and Mg^2+^ (0.065 nm) (Islam et al., 2022; Anjum et al., 2017). At an annealing temperature of 400°C, similar variations occur, although all six peaks are noticeable for *x ≥* 0.3. At the annealing temperatures of 600°C and 800°C, all the peaks are clearly visible with narrower peak widths, indicating grain growth and higher crystallinity right from *x =* 0. The peaks have shifted towards the lower angle side, indicating an increase in lattice spacing due to the grain growth. The nanoparticles possess a high ratio of surface area to volume. As the particle size increases, the core of the particles increases, and the surface atoms reduce. Consequently, the ordered phase increases, replacing an incomplete coordination of disordered surface atoms. This results in a peak shift as the crystallinity and grain growth increase (Fouad et al., 2019).

**FIGURE 2:**
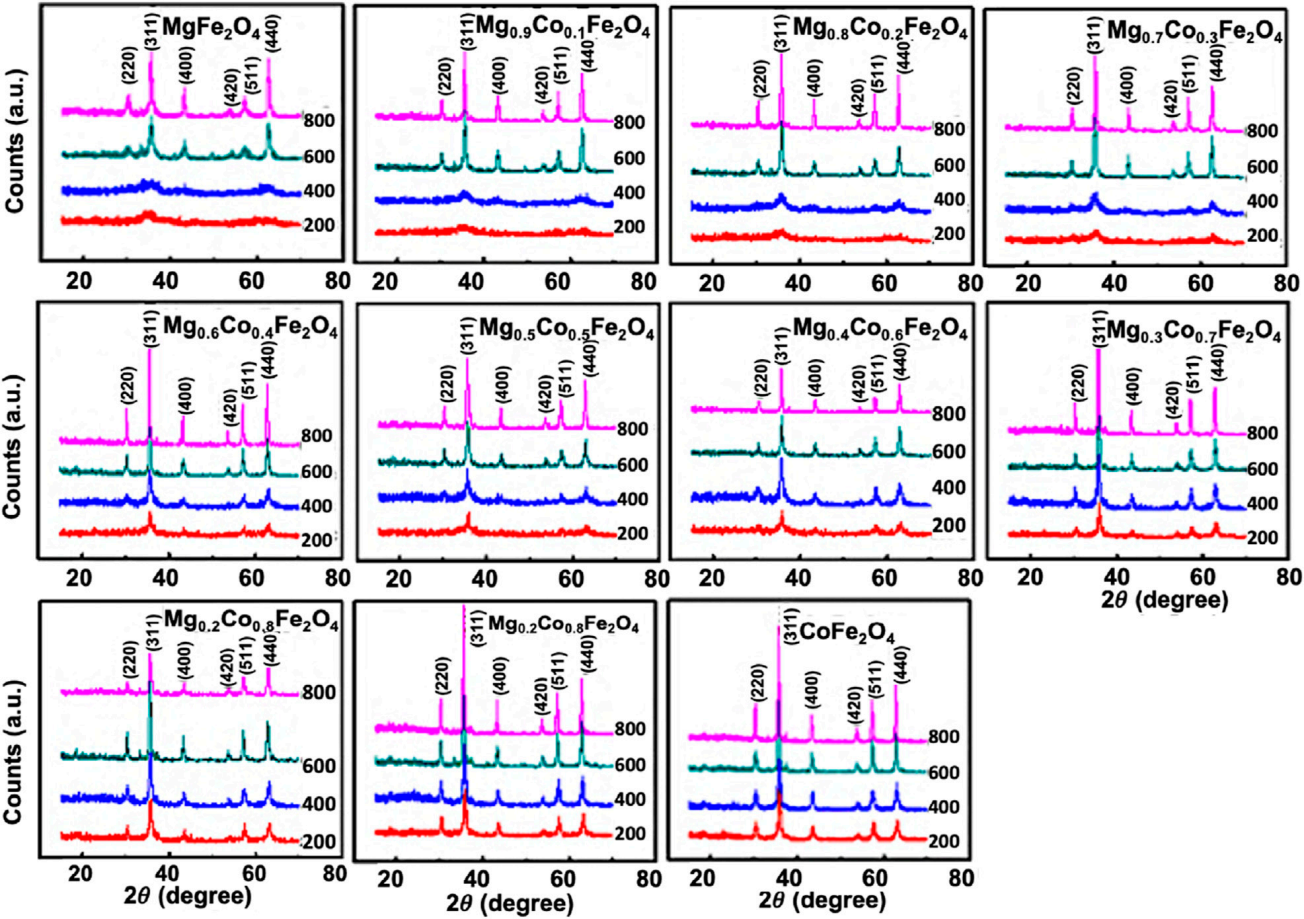
X-ray diffraction pattern of Mg_1*−x*_Co_*x*_Fe_2_O_4_ (where 0 ≤ x ≤ 1; ∆x = 0.1) nanoparticles annealed at 200°C, 400°C, 600°C, and 800°C. The XRD scan was performed on the powder samples, 2*θ* angle ranging from 15°–70° with a scan step size of 0.0167^o^.

The peak width of the diffraction peaks estimates the crystallite size. The kinematical theory of x-ray diffraction reveals that the total intensity of each diffraction peak from a single crystal, which relates to the scattering angle, *2θ,* is directly proportional to the volume of the crystal (Valério and Morelhao 2019). As the volume of the crystallites increases, the peak area also increases while the peak width (FWHM) *β* decreases. The width of the peak is inversely proportional to the size of the crystallite. The width of each peak ascertains the mean particle size of the crystallites. It is common practice to utilize the width of the maximum intensity peak, which has the highest multiplicity factor. In the current study, the peak of Miller indices (311) exhibits the highest multiplicity factor, resulting in the maximum intensity we used to determine the crystallite size. Figure 2 demonstrates that the peak width decreases as the crystallinity increases due to a concurrent rise in Co concentration and annealing temperature. Crystallinity and grain growth also result in a displacement of the peaks. This is because smaller particle size results in larger surface areas. When the crystallinity increases, the surface area decreases due to the coalescing of smaller grains and the increased coordination number, while the lattice strain decreases. By altering the lattice spacing, d, the position of the peak changes following Bragg’s Law.

We used Eq. 1 to determine the grain size where D is the average crystallite size, *λ* is the X-ray wavelength, *β* is the full width at half maximum value of the highest intensity (311) peak in radians, and *θ* is the Bragg angle (Kumar et al., 2013).

$$D=\frac{0.94\lambda }{\beta \cos\theta }$$ (1)

**FIGURE 3:**
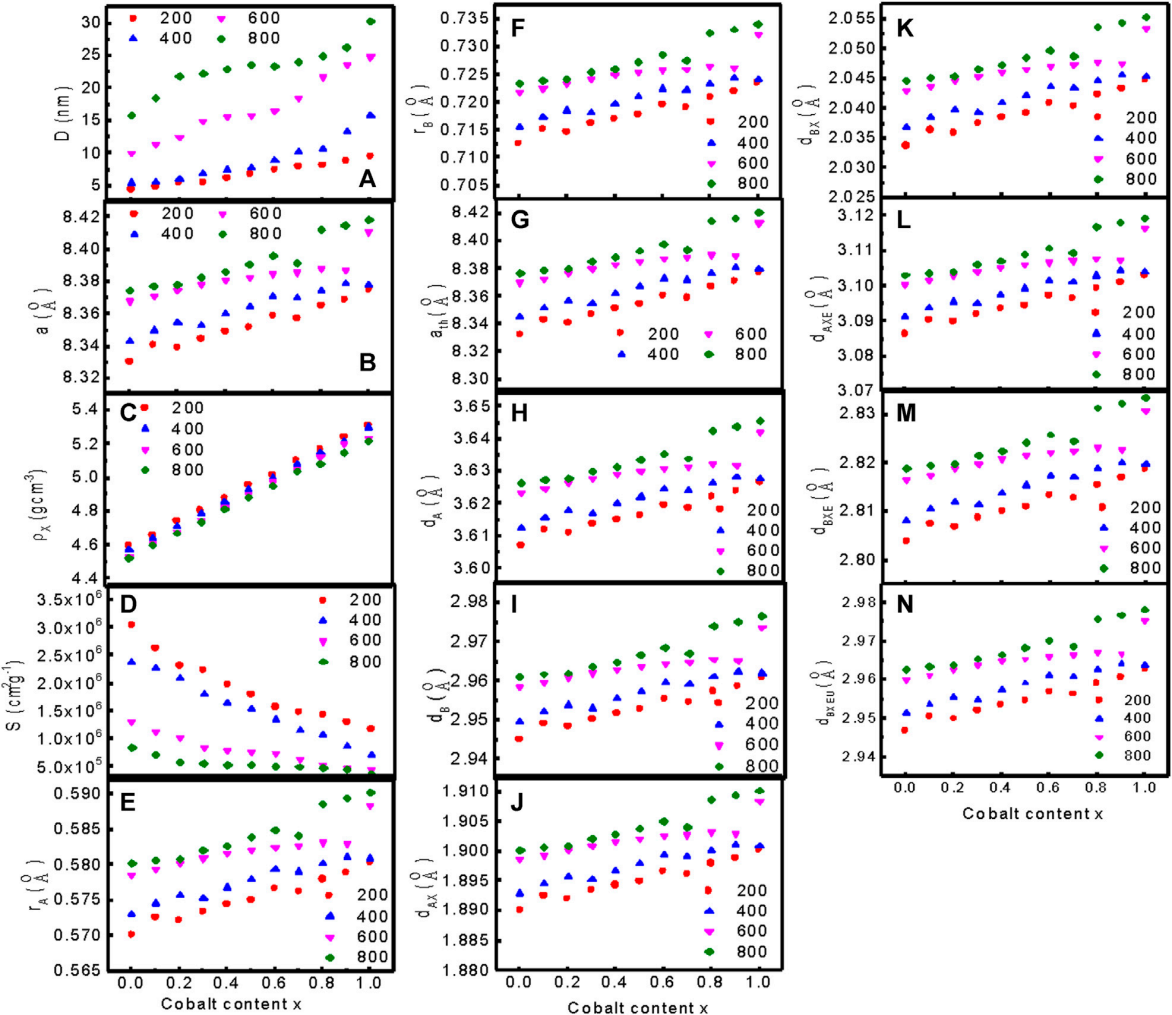
Variation of **(A)** the particle size D, **(B)** the observed lattice parameter a, **(C)** the X-ray density, **(D)** the specific surface area of the particles S, **(E)** the ionic radius of the tetrahedral site r_*A*_, **(F)** the ionic radius of octahedral site r_*B*_, **(G)** the theoretical lattice parameter *a*th, **(H)** the hopping length for tetrahedral site d_*A*_, **(I)** the hopping length for octahedral site d_*B*_, **(J)** the bond length of tetrahedral site d_*AX*_, **(K)** the bond length of octahedral site d_*BX*_, **(L)** the tetrahedral edge d_*AXE*_, **(M)** the shared octahedral edge d_*BXE*_, and **(N)** the unshared octahedral edge d_*BXEU*_ of MCFO nanoparticles at different values of x annealed at 200°C, 400°C, 600°C, and 800°C.

Figure 3 presents the variations of (a) the particle size D, (b) the observed lattice parameter a, (c) the X-ray density, (d) the specific surface area of the particles S, (e) the ionic radius of the tetrahedral site r_*A*_, (f) the ionic radius of octahedral site r_*B*_, (g) the theoretical lattice parameter *a*th, (h) the hopping length for tetrahedral site d_*A*_, (i) the hopping length for octahedral site d_*B*_, (j) the bond length of tetrahedral site d_*AX*_, (k) the bond length of octahedral site d_*BX*_, (l) the tetrahedral edge d_*AXE*_, (m) the shared octahedral edge d_*BXE*_, and (n) the unshared octahedral edge d_*BXEU*_ of MCFO nanoparticles at different values of *x* and annealed samples. Supplementary Table S1–S4 present the relevant data.

Figure 3A represents the variation of particle size with Co^2+^ content, *x* of MCFO ferrite nanoparticles annealed at different temperatures. Particle size increases with an increase in *x* and annealing temperature. The observed lattice parameter for each plane was calculated using Bragg’s law presented in Eq. 2 Kumar et al. (2013).

$$d_{hkl=\frac{a}{\sqrt{h^{2}+k^{2}+l^{2}}}}$$ (2)

Figure 3A presents the precise values of the lattice parameters determined using the Nelson-Riley function, F (θ). The intercept of the linear extrapolation of *a* vs. *F* (*θ*) for each plane gives the precise value of the lattice parameter (Cullity 2014; Chintala et al., 2021).

Figure 3B shows that the lattice parameter increases linearly with the increase in *x* and annealing temperature, which indicates that the dependence of the lattice parameter with composition follows Vegard’s Law (Denton and Ashcroft, 1991; Yadav et al., 2017). The lattice parameter increases with an increase in Co^2+^ content because the ionic radius of the Mg^2+^ (0.065 nm) ion is smaller than that of the Co^2+^ (0.072 nm) ion (Islam et al., 2022; Abraham et al., 2018). The lattice parameter also increases with an increase in annealing temperature because the core of the grains increases, replacing the disordered surface atoms with an increase in annealing temperature, and surface tension decreases (Li et al., 2020).

Figure 3C represents the variation of X-ray density with Co^2+^ content, *x* of MCFO nanoparticles annealed at different annealing temperatures. The X-ray density increases with an increase in *x* and annealing temperature. We calculated the specific surface area of the particles (S), the radius of the tetrahedral site (r_*A*_), the radius of the octahedral site (r_*B*_), the theoretical lattice parameter (*a*th), the hopping length for the tetrahedral site (d_*A*_), hopping length for the octahedral site (d_*B*_), the tetrahedral and the octahedral bond length(d_*AX*_ and d_*BX*_), the tetrahedral edge (d_*AXE*_), and the shared and unshared octahedral edge (d_*BXE*_ and d_*BXEU*_) for cubic spinel ferrite nanoparticles using the equation given in the literature (Satalkar and Kane, 2016). We used the equations in the supplementary section and Supplementary Table S1–S4 present all data at different compositions and annealing temperatures. Figures 3D–N shows the variation of S, r_*A*_, r_*B*_, d_*A*_, d_*B*_, d_*AX*_, d_*BX*_,d_*AXE*_, d_*BXE*_, and d_*BXEU*_ with Co^2+^ content, *x* of MCFO nanoparticles annealed at different annealing temperatures. The value of specific surface area, S, decreases with Co^2+^ content, *x*, and annealing temperature. This is expected because, when particle size increases with *x* and annealing temperatures, the specific surface area decreases since the core of the nanoparticles grows at the expense of disordered surface atoms. The value of r_*A*_, r_*B*_, d_*A*_, d_*B*_, d_*AX*_, d_*BX*_, d_*AXE*_, d_*BXE*_, and d_*BXEU*_ increases with an increase in Co^2+^ content and annealing temperature because particle size increases with an increase in Co^2+^ content and annealing temperature, which is associated with cation redistribution with grain growth. Co^2+^ has a tendency to occupy B-site, while Mg^2+^ ion has the tendency to occupy A-site. Changes in composition and annealing temperatures cause changes in cation distribution, which lead to the change in tetrahedral and octahedral radii, hopping length at the tetrahedral and octahedral sites, the bond length, and shared and unshared tetrahedral and octahedral edges.

**FIGURE 4:**
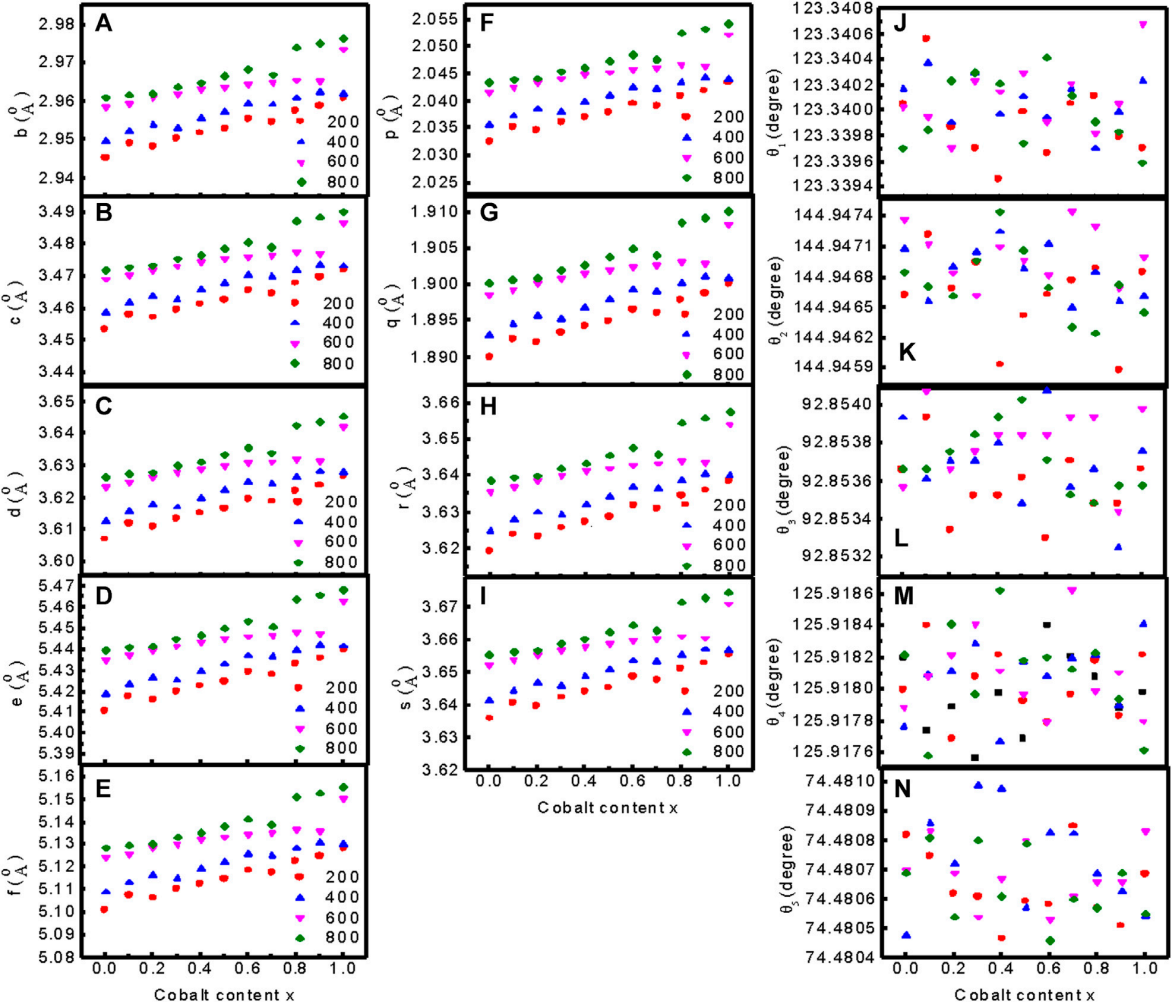
Variation of **(A–E)** the interionic distances between the cations b, c, d, e, and f, **(F–I)** the cation-anion distance p, q, r, and s, and **(J–N)** the bond angles θ_1_, θ_2_, θ_3_, θ_4_, and θ_5_ of Mg_1*−x*_Co_*x*_Fe_2_O_4_ nanoparticles annealed at 200°C, 400°C, 600°C, and 800°C.

Figures 4A–E presents the variation of the interionic distances between the cations, (f–i) the cation-anion distance, and (j–n) the bond angle of Mg_1*−x*_Co_*x*_Fe_2_O_4_ nanoparticles annealed at 200°C, 400°C, 600°C, and 800°C. Figures 4A–E presents the interionic distances between cations *b, c, d, e,* and *f* obtained using the equation presented in the literature by (Satalkar and Kane, 2016). We presented all the equations in the supplementary section and the deduced data in Supplementary Table S5–S8. Figures 4F–I present the spaces between cations and anions *p, q, r,* and *s* using the equations in the supplementary section and the deduced data in Supplementary Table S5–S8. The cation-cation distances and cation-anion distances increase with an increase in Co^2+^ content and annealing temperature because the ionic radius of Co^2+^ (0.072 nm) is higher than that of Mg^2+^ (0.065 nm). Figures 4J–N show the variations in bond angles *θ*_1_, *θ*_2_, *θ*_3_, *θ*_4_, and *θ*_5_ at different compositions and annealing temperatures. Change in cation distribution with composition and annealing temperature also leads to change in the cation-cation, cation-anion distances, and the bond angles.

#### 3.1.2 Transmission electron microscopy (TEM)

**FIGURE 5:**
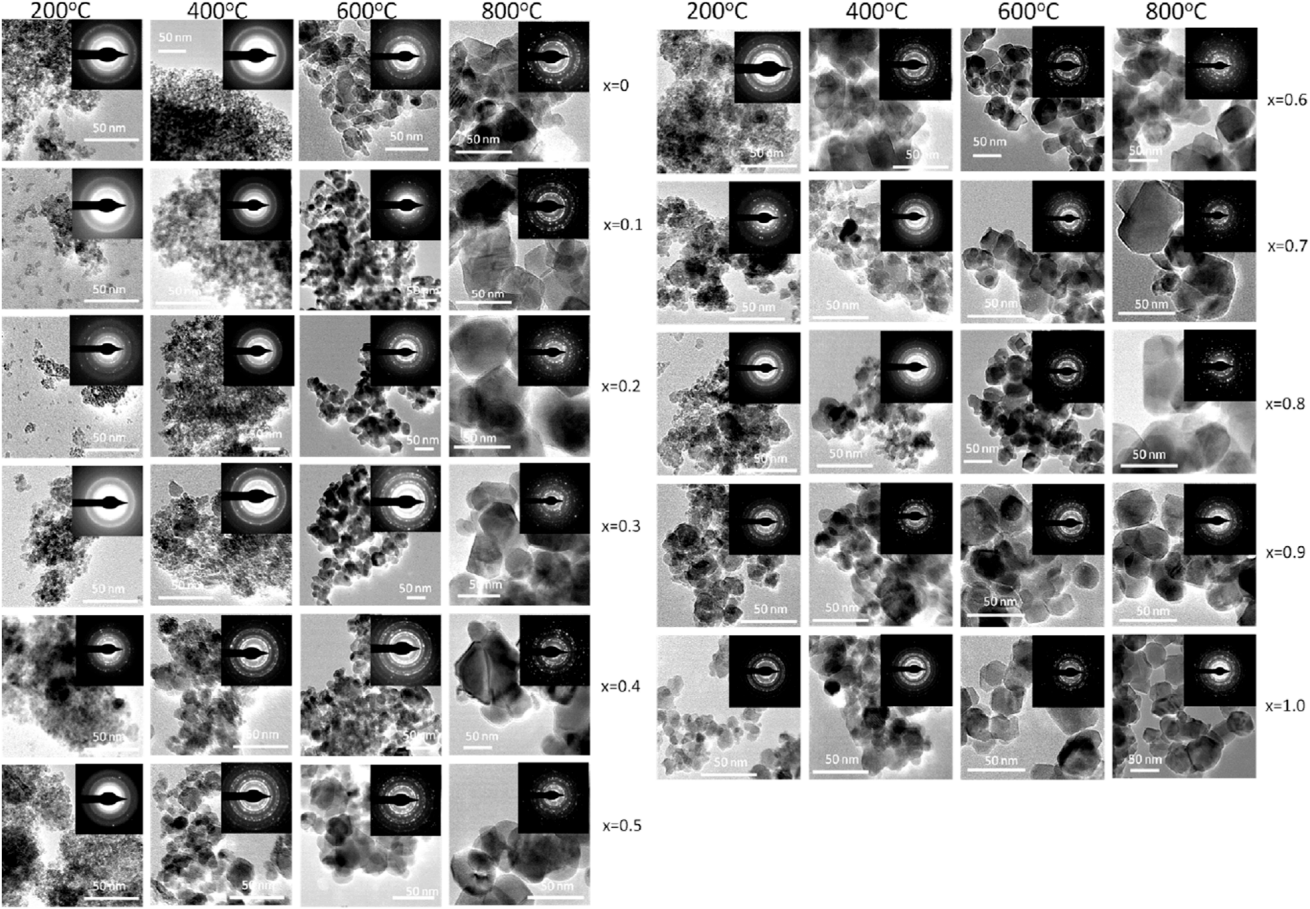
TEM image of Mg_1*−x*_Co_*x*_Fe_2_O_4_ nanoparticles (where 0 ≤ x ≤ 1; ∆x = 0.1) annealed at 200°C, 400°C, 600°C, and 800°C. TEM images were acquired from drop cast samples on carbon-coated Cu grid. Particle size increases with increasing cobalt content x and annealing temperature. In the inset, selected area electron diffraction patterns (SAED) are presented.

**FIGURE 6:**
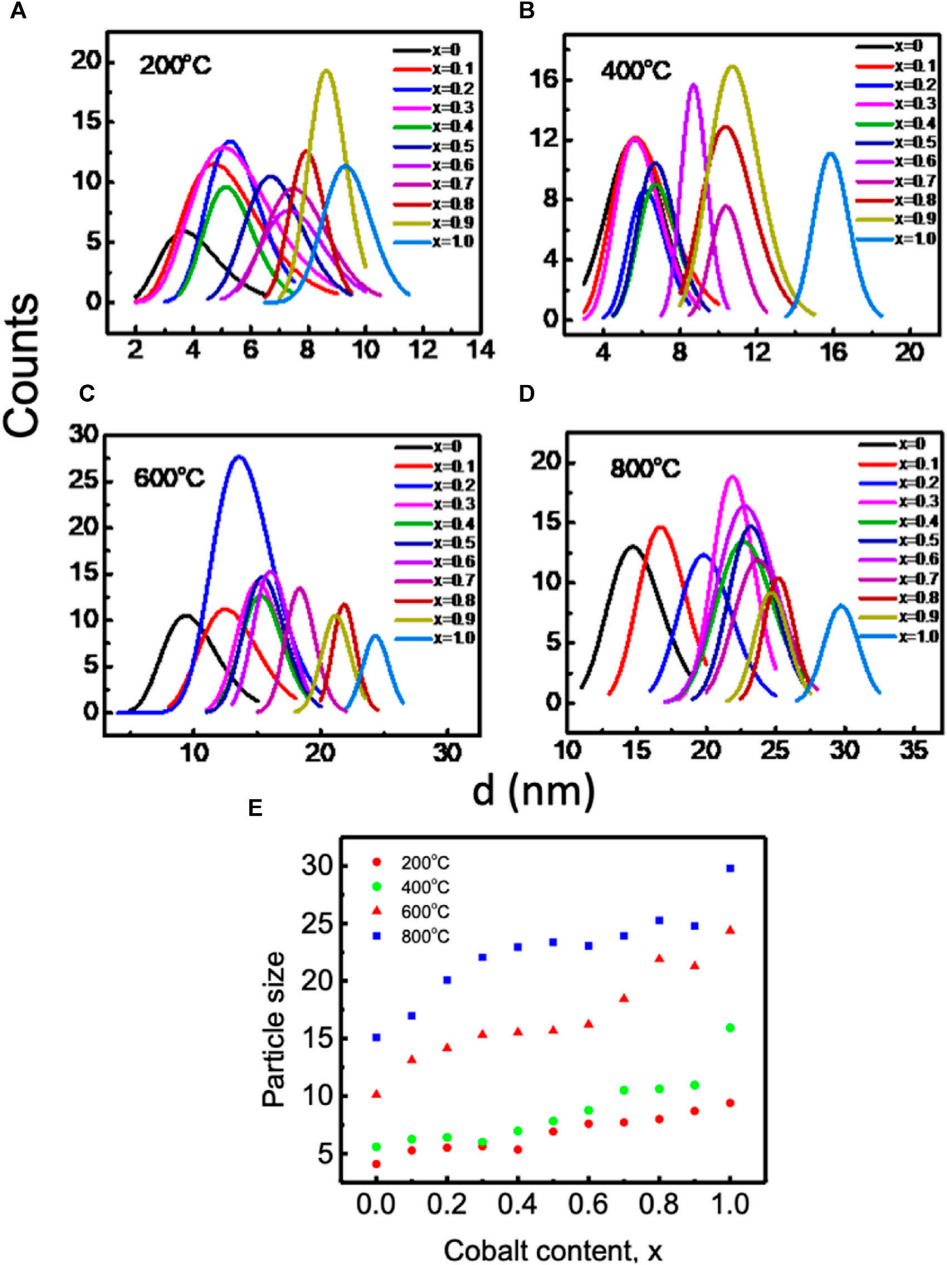
Lognormal distribution of Mg_1_-_*x*_Co_*x*_Fe_2_O_4_ (where 0 ≤ x ≤ 1; ∆x = 0.1) nanoparticles annealed at **(A)** 200°C, **(B)** 400°C, **(C)** 600°C, and **(D)** 800°C and **(E)** variations of average particle size acquired from **(A)** to **(D)** with cobalt content and annealing temperatures. Particle size increases with increasing cobalt content and annealing temperature.

Figures 5A,B displays transmission electron microscopy (TEM) images of Mg_1−x_Co_x_Fe_2_O_4_ nanoparticles, where x ranges from 0 to 1 with an increment of 0.1. The nanoparticles were annealed at temperatures of 200, 400C, 600, and 800°C. The inset displays selected area diffraction (SAED) patterns, exhibiting the most prominent peak (311) as well as additional planes such as (220), (400), (420), (511), and (440). The d_hkl_ values in the SAED patterns were determined using Velox software, and the corresponding diffractograms were indexed accordingly. In addition, SAED patterns also indicate that the Debye rings are wide for smaller particle sizes. The sharpness of the rings increases as the crystallinity and particle size increase. The TEM images exhibit semi-spherical particles that exhibit grain growth as the cobalt concentration x and annealing temperatures increase. The particle size distributions for all annealing temperatures and compositions were determined by analyzing the histogram of the size distribution, which exhibits a log-normal distribution. Figures 6A–D displays the log-normal distribution of MCFO nanoparticles and Figure 6E presents the relationship between particle size and composition, as obtained from the histogram. The data also indicates that the particle size tends to rise as the cobalt content and annealing temperature increase. Figure 3A depicts the relationship between the size of crystallite and x, obtained by X-ray diffraction, at various annealing temperatures. The particle sizes measured by X-ray diffraction (XRD) in Figure 3A and transmission electron microscopy (TEM) in Figure 6E are in good agreement.

**FIGURE 7:**
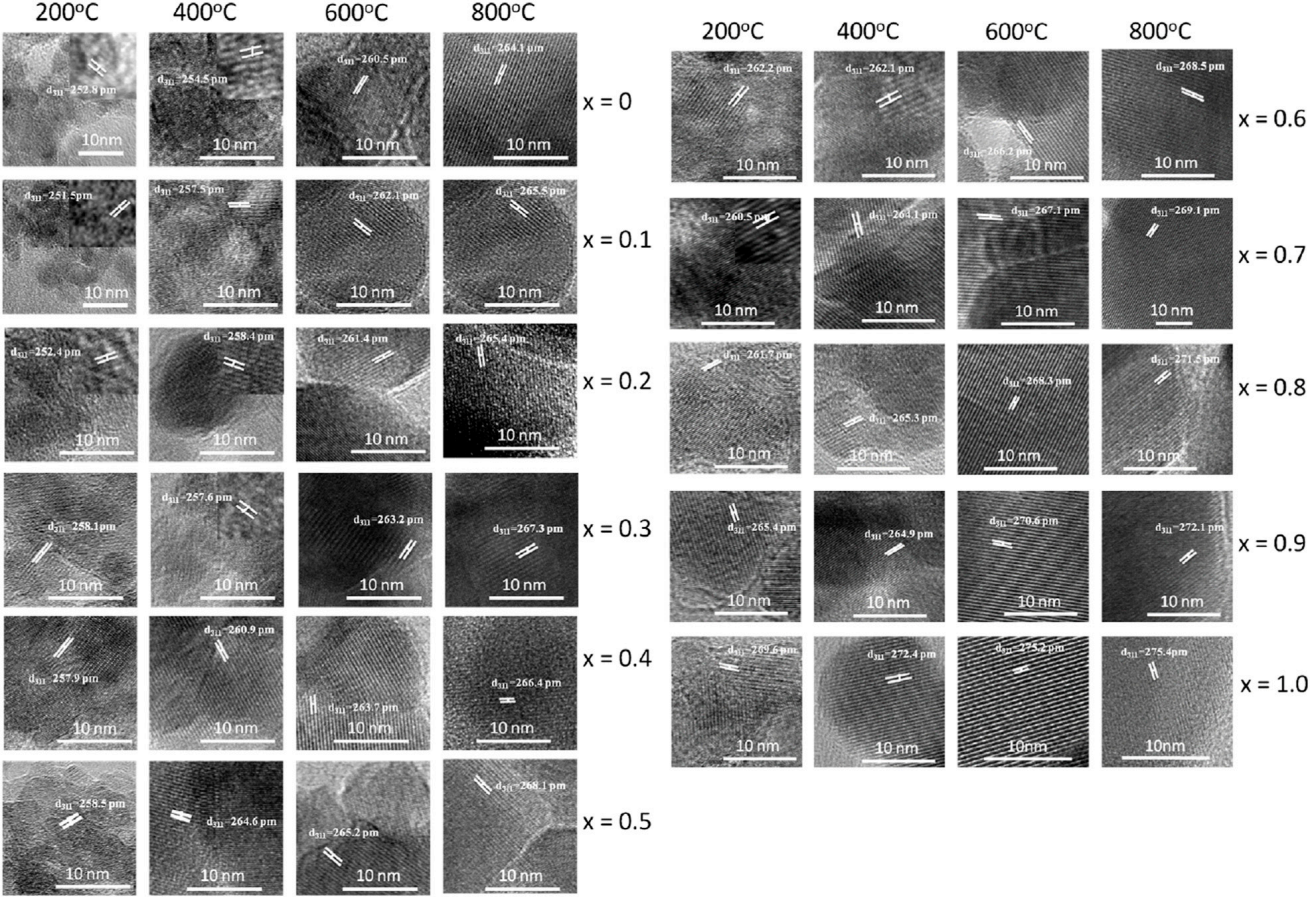
HRTEM images of Mg_1-*x*_Co_*x*_Fe_2_O_4_ nanoparticles (0*≤x ≤* 1; ∆*x* = 0.1) annealed at 200°C, 400°C, 600°C, and 800°C. In the figure, lattice spacings of the lattice fringes are marked. Lattice fringes are more pronounced with the increase of *x* and annealing temperature. Particle size increases with increasing cobalt content *x* and annealing temperature.

Figure 7 displays high-resolution transmission electron microscopy (HRTEM) images of MCFO nanoparticles subjected to different annealing temperatures: 200, 400, 600, and 800°C. The HRTEM images indicate that all the samples under investigation possess a nanocrystalline structure, with the degree of crystallinity being directly proportional to both the x and the annealing temperatures. MCFO with higher cobalt concentration and higher annealing temperatures exhibited distinctive lattice fringes in the HRTEM image, which result in a significant enhancement of crystallinity.

**FIGURE 8:**
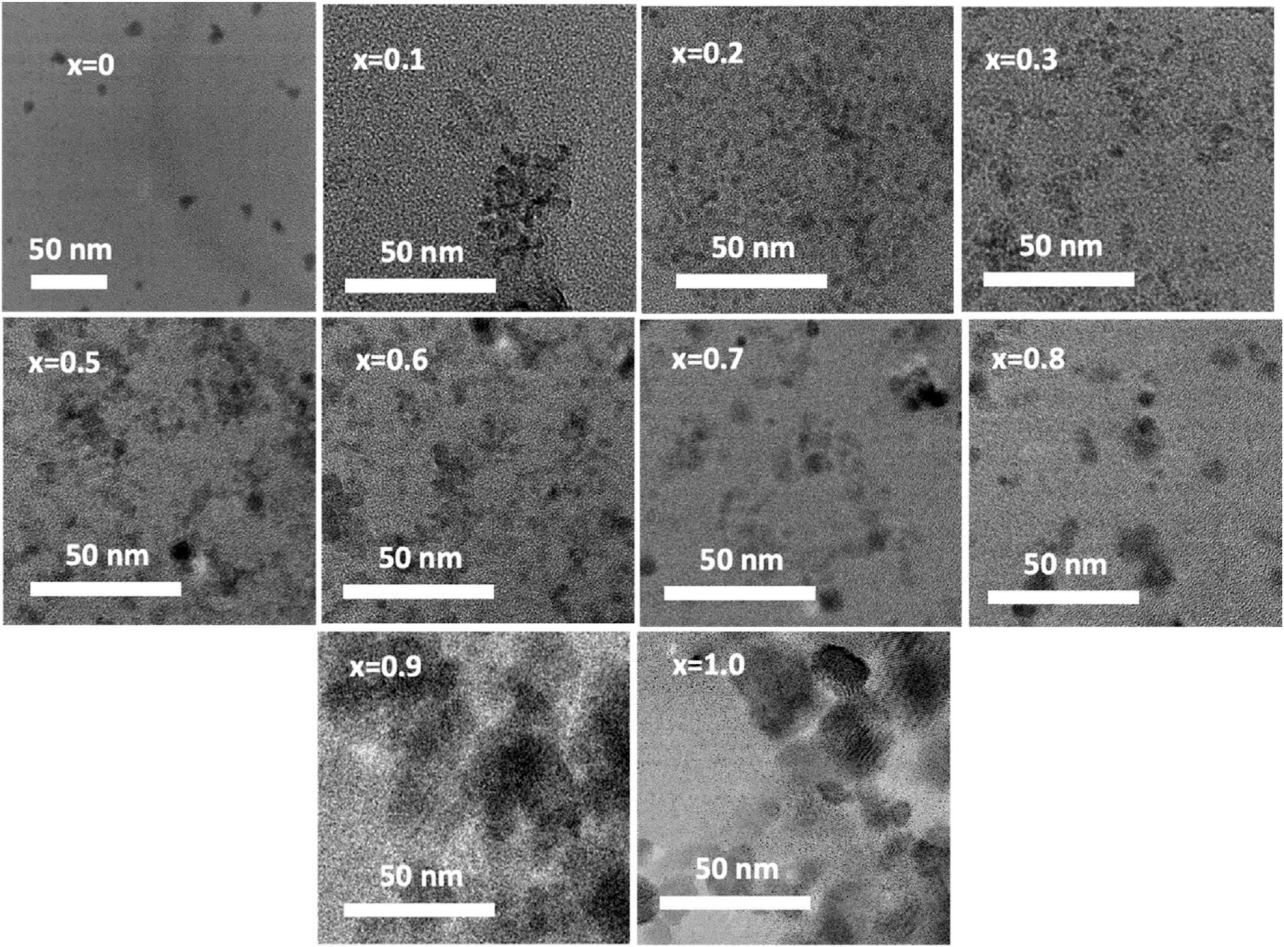
TEM images of chitosan-MCFO nanohybrids (Mg_1-*x*_Co_*x*_Fe_2_O_4_; 0*≤x ≤* 1; ∆*x* = 0.1). In this figure, MCFO nanoparticles were annealed at 200°C. The nanohybrids in this figure are more disperse compared with the images of the uncoated MCFO nanoparticles annealed at the same temperature in Figure 5. Uncoated samples are more agglomerated than the coated nanohybrids.

Figure 8 displays chitosan-MCFO nanohybrids with varying compositions (0 ≤ x ≤ 1; ∆x = 0.1) that have been subjected to annealing at a temperature of 200°C. The illustration demonstrates that the particles exhibit a higher degree of dispersion when they are coated with chitosan. These figures can be compared with the images depicted in Figure 5 of uncoated samples annealing at the same temperature of 200°C. The samples annealed at other temperatures are anticipated to exhibit a more comparable dispersion of chitosan-MCFO nanohybrid than the MCFO nanoparticles.

#### 3.1.3 Raman spectroscopy

**FIGURE 9:**
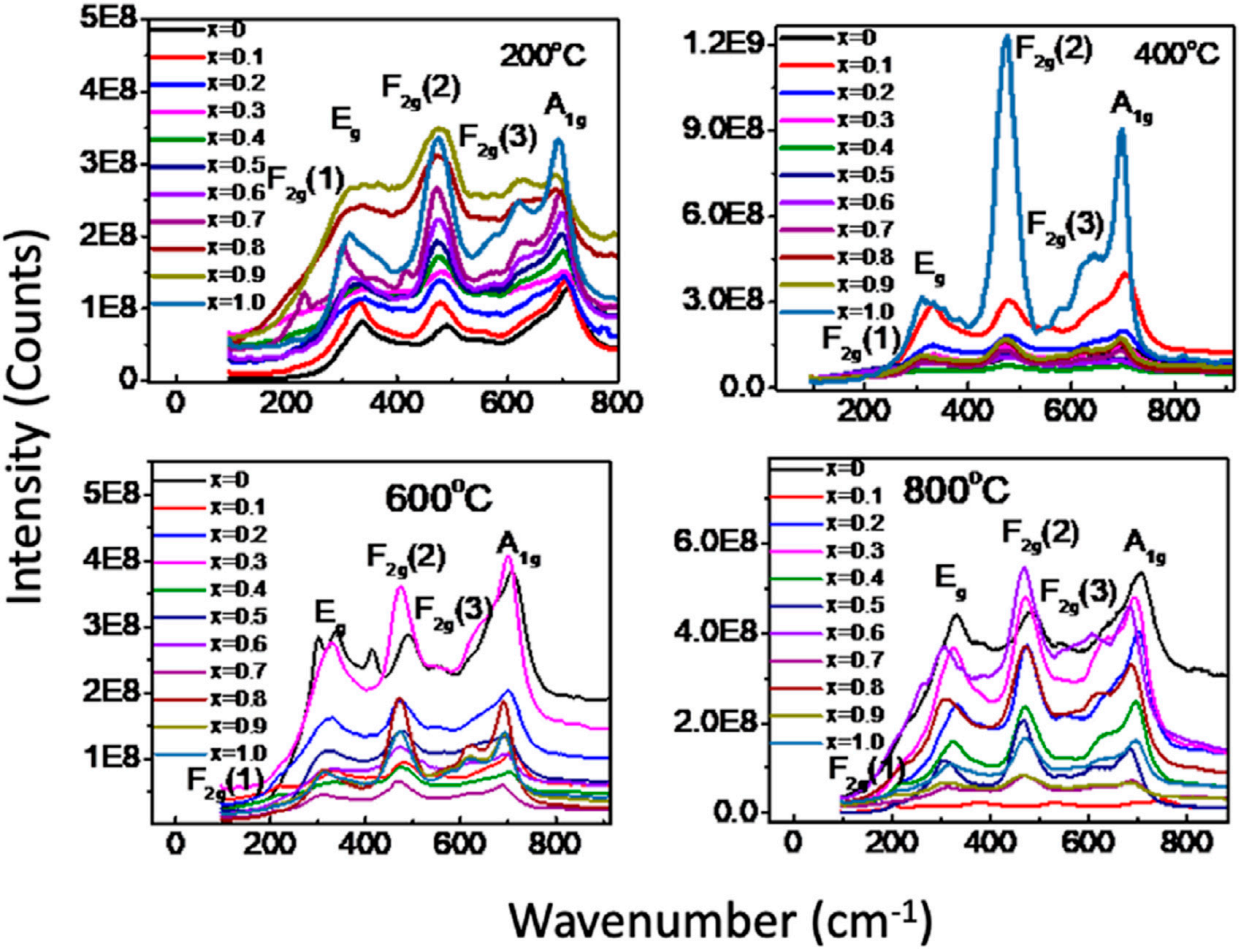
(a) Room temperature Raman spectra of Mg_1-*x*_Co_*x*_Fe_2_O_4_ nanoparticles (0*≤x ≤* 1; ∆*x* = 0.1) annealed at 200°C, 400°C, 600°C, and 800°C in the range of 190–1,000 cm^−1^ using the pelletized solid samples. Five Raman active modes A_1g_, E_g_, F_2g_(1),F_2g_(2), and F_2g_(3) are assigned in the Raman spectra according to the previous work.

To conduct a more detailed structure analysis, we employed Raman spectroscopy to examine MCFO nanoparticles subjected to annealing at 200°C–800°C to find vibrational modes. Figure 9 displays the Raman spectra of MCFO nanoparticles of annealed samples. The spectra were acquired in the range 190–1,000 cm^−1^ at room temperature. It was well-established in the literature that MCFO ferrites possess a partially inverse spinel structure belonging to the Fd−3m space group. The vibrational modes associated with this space group are A_1g_ (R), E_g_ (R), F_1g_, 3F_2g_ (R), 2A_2u_, 2E_u_, 4F_1u_ (IR), and 2F_2u_. The notation “R" indicates Raman active vibrational modes, “IR” represents infrared–active vibrational modes, and the other modes are silent. The Raman spectrum would exhibit the Raman active modes A_1g_, E_g_, F_2g_(3), F_2g_(2), and F_2g_(1) (Soler et al., 2004; Galinetto et al., 2018).

**FIGURE 10:**
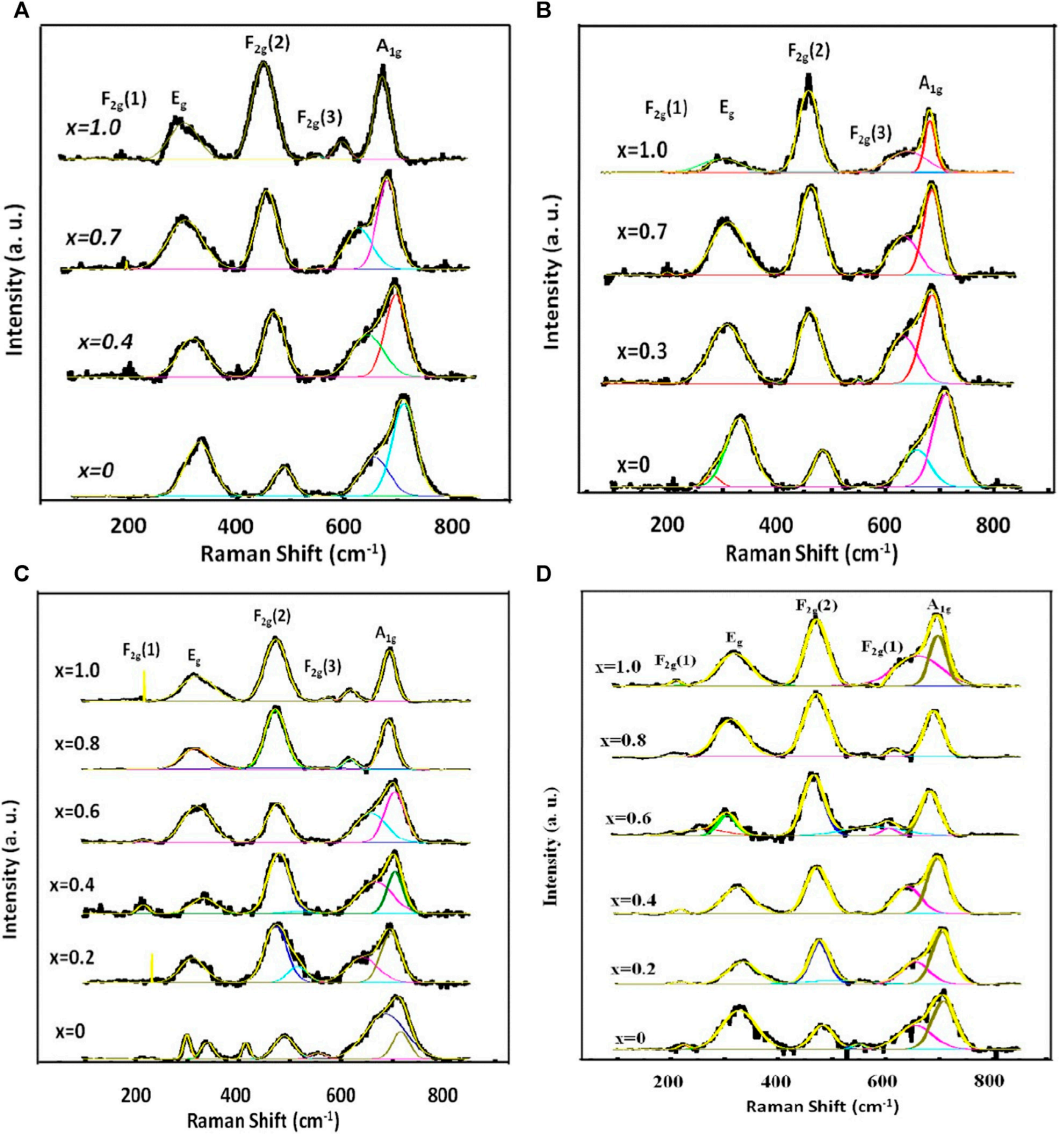
Representative curves that demonstrate the best Gaussian fitting of the experimental Raman spectra of Mg_1-*x*_Co_*x*_Fe_2_O_4_ nanoparticle (0 *≤ x ≤* 1; ∆*x* = 0.1) annealed at **(A)** 200, **(B)** 400, **(C)** 600 and **(D)** 800°C. The Gaussian fitting was performed with background subtraction and deconvolution operation.

Figure 10 illustrates the rendition of the Raman spectra of annealed nanoparticles achieved by applying the Gaussian function after subtracting the background and performing deconvolution. Gaussian function yielded the validation between the experimental and theoretical data. The A_1g_ modes correspond to the symmetric stretching of MeO_4_ (where Me represents Co and Mg) and FeO_4_ at the A-site. The E_g_ modes correspond to the bending of oxygen atoms with respect to the iron atom at the B-site. The F_2g_(3) modes correspond to the antisymmetric bending of oxygen in the presence of Fe while the F_2g_(2) modes depict the asymmetric stretching of Fe in conjunction with O. The F_2g_(1) modes exhibit the translational movement of both Fe and O. The Raman peaks within the range of 660–720 cm^−1^ correspond to the vibrational modes of the A-site, while the Raman peaks within the range of 460–660 cm^−1^ correspond to the vibrational modes of the B-site in the ferrites (Soler et al., 2004; Galinetto et al., 2018; Baig et al., 2021).

**FIGURE 11:**
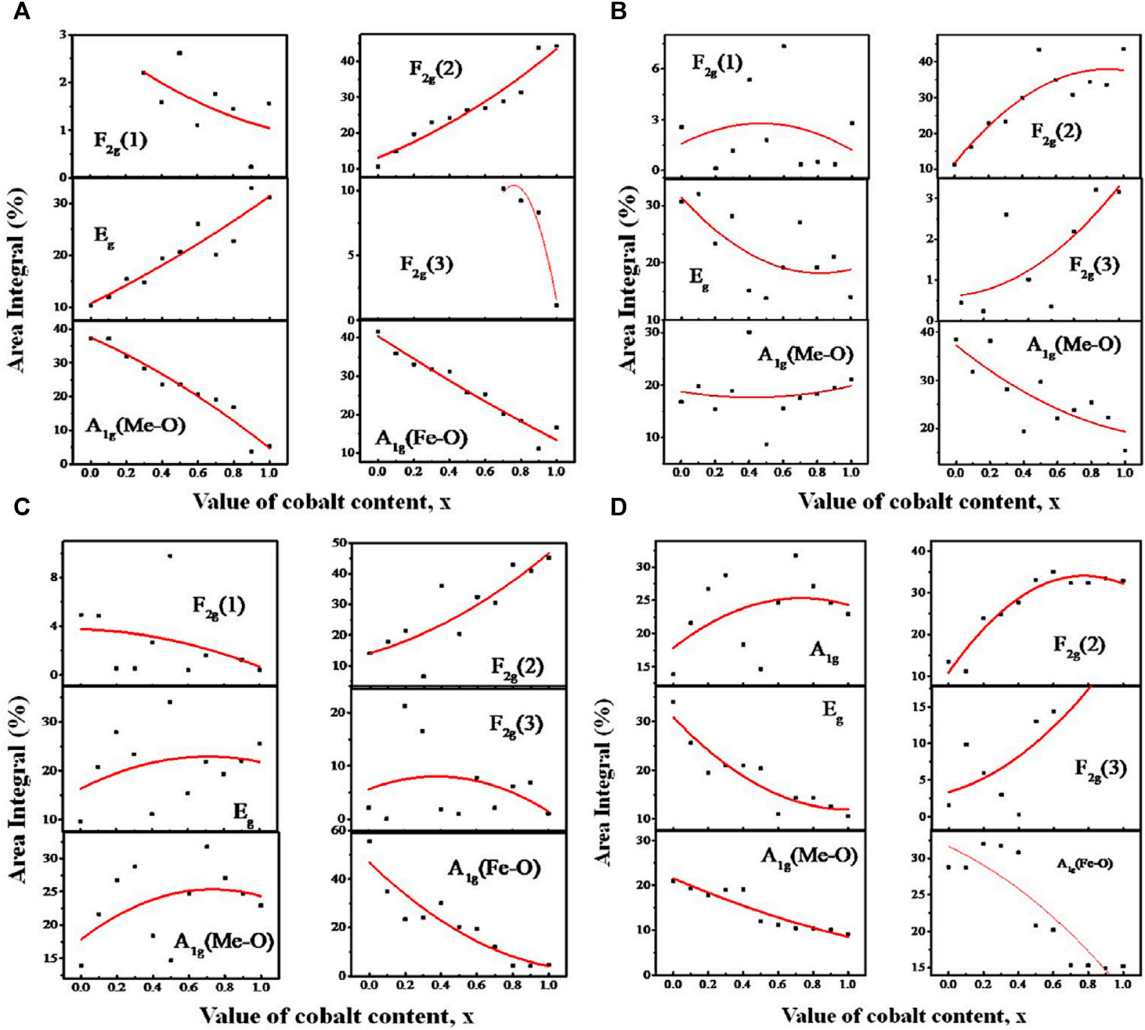
Variation of area integral with Co concentration *x* of the A_1g_, E_g_, F_2g_(1), F_2g_(2), and F_2g_(3) peaks assigned to the Raman spectra of MCFO nanoparticles in the previous figure, obtained by Gaussian fitting and deconvolution operation. In the Figure, the variation of the area integral for the samples annealed at **(A)** 200, **(B)** 400, **(C)** 600, and **(D)** 800°C are presented.

Figures 11A–D illustrate the relationship between the area integral and composition at the annealing temperatures ranging from 200 to *800*°*C*. The largest A-site occupancy for both Me and Fe in Figure 11A is observed for MgFe_2_O_4_ at an annealing temperature of 200°C, corresponding to a particle size range of 4.3–9.6 nm. The rise in Co content leads to a decrease in the occupancy of both Me (Mg and Co) and Fe on the A-site, while E_g_ and F_2g_(2) increase due to the increase in B-site occupancy. These findings indicate that for 4.3–9.6 nm particle size, compositions with a higher concentration of magnesium tend to favor the normal spinel structure, while compositions with a higher concentration of cobalt tend to favor the inverse spinel structure. The area integral of additional small intensity peaks, F_2g_(1) and F_2g_(3), remain constant regardless of the composition.

At 400°*C*, for particle size range 5.5–15.8 nm in Figure 11B, the relationship between the area integral A_1g_ and F_2g_(2) becomes relatively random with *x*. Nevertheless, there is a noticeable tendency of decreased A_1g_ and increased F_2g_(2) with *x*. This reflects a transformation of the spinel structure from normal to inverse spinel structure. In Figure 11C, the scattered nature of the area integral with composition is more pronounced for the particle size range of 10.1–24.9 nm at an annealing temperature of 600°*C*. Nevertheless, it was seen that the area integral of A_1g_ for Fe ions is dropping (A-site occupancy), while the area integral of F_2g_(2) is growing (B-site occupancy). This indicates a decrease in A-site occupancy and an increase in B-site occupancy with the increase of Co, resulting in a transition from a normal to an inverse spinel structure with *x*. At 800°C, specifically for particle sizes ranging from 15.9 to 30.3 nm, the occupancy of A-sites declines while B-sites increase with the increase of Co. This indicates a transition in the spinel structure from normal to inverse spinel with x. The Raman shift values for the Raman-active modes A_1g_, E_g_, F_2g_(1), F_2g_(2), and F_2g_(3) of the Mg_1−x_Co_x_Fe_2_O_4_ composition annealed at temperatures ranging from 200°C to 800°C are provided in Tables 1, Table 2, Table 3, and Table 4. These values were obtained using Gaussian fitting and deconvolution of the spectra shown in Figure 10. The frequencies of A_1g_ vibrational modes decrease when the cobalt content, x, increases in all samples. This is due to the substitution of lighter magnesium (24.3050 emu) ions with heavier cobalt (58.9332 amu) ions with the increase of Co^2+^. The Raman shift of F_2g_(2) also exhibits a reduction as the Co^2+^ increases. In this case also, magnesium (24.3050 amu) ions are replaced with the heavier cobalt (58.9332 amu) ions, which have a greater atomic mass. To summarize, particles with higher magnesium content have a more pronounced normal spinel structure. Conversely, particles containing higher amounts of cobalt display a more pronounced inverse spinel structure. Raman shift basically decreases at all values of x for both A and B sites because of the greater atomic mass Co^2+^ than Mg^2+^.

**TABLE 1:** Wavenumbers of the five Raman active modes A_1g_, E_g_, F_2g_(1),F_2g_(2), and F_2g_(3) of nanoparticles annealed at 200°C assigned to the Raman spectra.

x	Main Raman mode peak energy (cm^−1^)					
	F_2g_(1)	E_g_	F_2g_(2)	F_2g_(3)	A_1g_ (Me-O)	A_1g_ (Fe-O)
0	-	330	487	-	656	711
0.1	-	327	478	-	673	707
0.2	-	327	477	-	675	706
0.3	210	341	480	-	674	706
0.4	208	326	476	-	651	703
0.5	207	325	475	-	653	700
0.6	214	323	475	-	645	698
0.7	181	323	474	553	642	697
0.8	174	323	472	-	638	692
0.9	146	322	474	545	621	691
1	164	325	473	570	618	690

**TABLE 2:** Wavenumbers of the five Raman active modes A_1g_, E_g_, F_2g_(1), F_2g_(2), and F_2g_(3) of nanoparticles annealed at 400°C assigned to the Raman spectra.

x	Main Raman mode peak energy (cm^−1^)					
	F_2g_(1)	E_g_	F_2g_(2)	F_2g_(3)	A_1g_ (Me-O)	A_1g_ (Fe-O)
0	281	331	484	-	657	711
0.1	-	330	479	-	652	706
0.2	162	333	479	-	645	702
0.3	142	323	476	564	648	702
0.4	214	334	480	581	664	705
0.5	127	336	479	591	653	697
0.6	182	333	476	566	642	695
0.7	208	320	472	563	644	696
0.8	210	320	471	524	640	694
0.9	243	317	473	490	646	697
1	215	319	471	574	618	693

**TABLE 3:** Wavenumbers of the five Raman active modes A_1g_, E_g_, F_2g_(1),F_2g_(2), and F_2g_(3) of nanoparticles annealed at 600°C assigned to the Raman spectra.

x	Main Raman mode peak energy (cm^−1^)					
	F_2g_(1)	E_g_	F_2g_(2)	F_2g_(3)	A_1g_ (Me-O)	A_1g_ (Fe-O)
0	298	338	488	554	686	715
0.1	219	331	481	589	677	712
0.2	213	318	475		657	704
0.3	200	325	466	480	651	702
0.4	213	331	478	519	666	704
0.5	205	317	474	553	650	698
0.6	229	310	472	514	641	696
0.7	212	313	470	507	629	687
0.8		314	471	476	617	691
0.9	198	316	471	535	616	691
1	215	319	471	574	618	693

**TABLE 4:** Wavenumbers of the five Raman active modes A1g, Eg, F2g(1),F2g(2), and F2g(3) of nanoparticles annealed at 800°C assigned to the Raman spectra.

x	Main Raman mode peak energy (cm^−1^)					
	F2g(1)	Eg	F2g(2)	F2g(3)	A1g (Me-O)	A1g (Fe-O)
0	224	337	482	550	655	708
0.1	202	331	479	549	654	707
0.2	216	331	474	505	653	703
0.3	216	329	474	532	653	703
0.4	216	324	472	548	640	697
0.5	204	311	465	486	626	685
0.6	263	309	469	580	609	685
0.7	208	317	471		607	690
0.8	208	317	471		607	690
0.9	206	311	468		626	686
1	206	310	467	579	626	685

#### 3.1.4 Fourier-transform infrared spectroscopy (FTIR)

**FIGURE 12:**
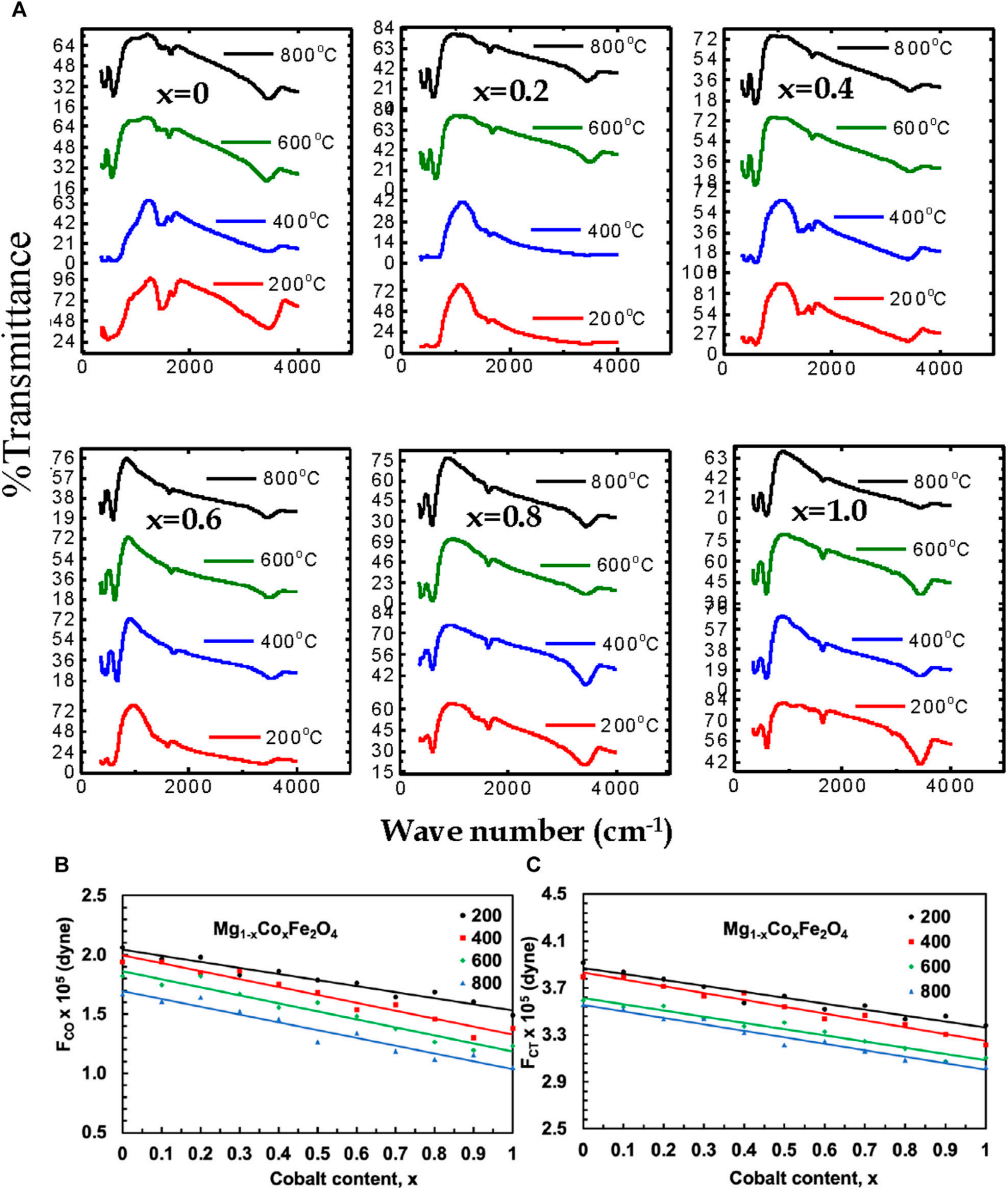
**(A)** The FTIR spectra of Mg_1-*x*_Co_*x*_Fe_2_O_4_ nanoparticles (0 *≤ x ≤* 1; ∆*x* = 0.1) annealed at 200°C, 400°C, 600°C, and 800°C, **(B)** the variation of the octahedral force constant F_CO_, and **(C)** the variation of tetrahedral force constant F_CT_ with cobalt content *x* are presented for the annealed samples.

Fourier-transform infrared spectroscopy (FTIR) of MCFO nanoparticles annealed at 200°C, 400°C, 600°C, and 800°C are presented in Figure 12. Absorption peaks at 3,430–3,493 cm^−1^ were observed due to the stretching vibration of the O-H group (Zeeshan et al., 2018). Two characteristic peaks of spinel ferrite of the cubic structure at the lower frequency region were observed. The higher frequency band is related to the stretching vibration of a metal-oxide bond at the tetrahedral (A) site and the lower frequency band is related to the stretching vibration of a metal-oxide bond at the octahedral (B) site because the bond length of the A site is shorter than the bond length of the B site (Zeeshan et al., 2018; Naseri et al., 2014; Gadkari et al., 2009; Pawlak and Mucha, 2003; Josyulu and Sobhanadri, 1981; Mund and Ahuja, 2016; Sharma et al., 2016). The frequency of both the higher frequency band and the lower frequency band shifted towards the higher frequency region with an increase in Co^2+^ content because Co^2+^ (0.072 nm) replaces Mg^2+^ (0.065 nm) at the A site and Fe^2^+ (0.0645 nm) at the B site which extends the covalent bond. Both the frequency bands shifted toward the higher frequency region with increasing annealing temperatures because particle size increases with an increase in annealing temperature.

Magnesium-rich compositions prefer normal spinel structures in their Raman spectroscopy, whereas cobalt-rich compositions demonstrate a preference for mixed spinel structures. Raman and FTIR spectroscopy produce similar outcomes. The reason is that the vibrational modes of the inverse spinel structure exhibit more clarity compared to those of the normal spinel structure, making them more readily detectable using Raman spectroscopy. FTIR spectroscopy can detect a wider variety of vibrational modes, which allows it to accurately identify both inverse and normal spinel structures. Consequently, these two methods can be employed in conjunction to discern and distinguish various spinel configurations. Additionally, they aid in the characterization of the vibrational modes of A-site and B-site at different compositions and annealing temperatures. At a temperature of 200°C, an absorption band with a lower frequency (v_1_) was detected in the range of 403–418 cm^−1^, while an absorption band with a higher frequency (ν_2_) was found in the range of 603–622 cm^−1^ for various compositions. The lower frequency absorption band (ν_1_) was detected at a temperature of 400°C, with a range of 398–415 cm^−1^. Similarly, the higher frequency absorption band (ν_2_) was identified within the range of 597–618 cm^−1^. The absorption band (ν_1_) with lower frequency, ranging from 395 to 412 cm^−1^, was seen at a temperature of 600°C. Additionally, the absorption band (ν_2_) with higher frequency, ranging from 593 to 610 cm^−1^, was also observed at the same temperature. Finally, at a temperature of 800°C, the absorption band with a lower frequency (ν_1_) was detected in the range of 391–408 cm^−1^, while the absorption band with a higher frequency (ν_2_) was seen in the range of 580–600 cm^−1^. The frequency band changed towards the lower frequency region as the particle size (annealing temperature) increased, due to the contraction of the covalent bond with larger particle sizes (annealing temperatures). Smaller particles exhibit a greater surface-to-volume ratio, which leads to incomplete coordination. In contrast, larger particles have a smaller surface area, resulting in more complete coordination and causing covalent bonds to decrease.

The force constants (FC) for the A site (F_CT_) and B site (F_CO_) are obtained using the following relation:

$$F_{C}=4\pi^{2}c^{2}\nu^{2}m$$ (4)

where c is the speed of light, v is the vibration frequency of the A site and B site, and m is the reduced mass for the Fe^2^+and O^2−^ ions (Yadav et al., 2017). Variations of F_*CT*_ and F_*CO*_ of Mg_1*−x*_Co_*x*_Fe_2_O_4_ ferrites annealed at 200°C, 400°C, 600°C, and 800°C with Co^2+^ content, x are presented in Figure 12B,C. The data are presented in Supplementary Table S9. The F_*CT*_ and F_*CO*_ increases with increasing Co^2+^ content x because the bond length of the A site and the B site decreases with an increase in Co^2+^content (Rana et al., 2010). In our previous study, Islam et al. (2022), FTIR spectra of uncoated and chitosan-coated MCFO in the as-dried condition are presented. The good bond of chitosan and MCFO is manifested from the peak shift of A and B-site bonding. FTIR spectra of uncoated and chitosan-coated MCFO in the as-dried condition are presented. The chitosan and MCFO bonded well, which was manifested in the peak shift of A and B-site bonding before and after coating. We expect a similar situation in the present study also.

#### 3.1.5 Magnetization measurements

**FIGURE 13:**
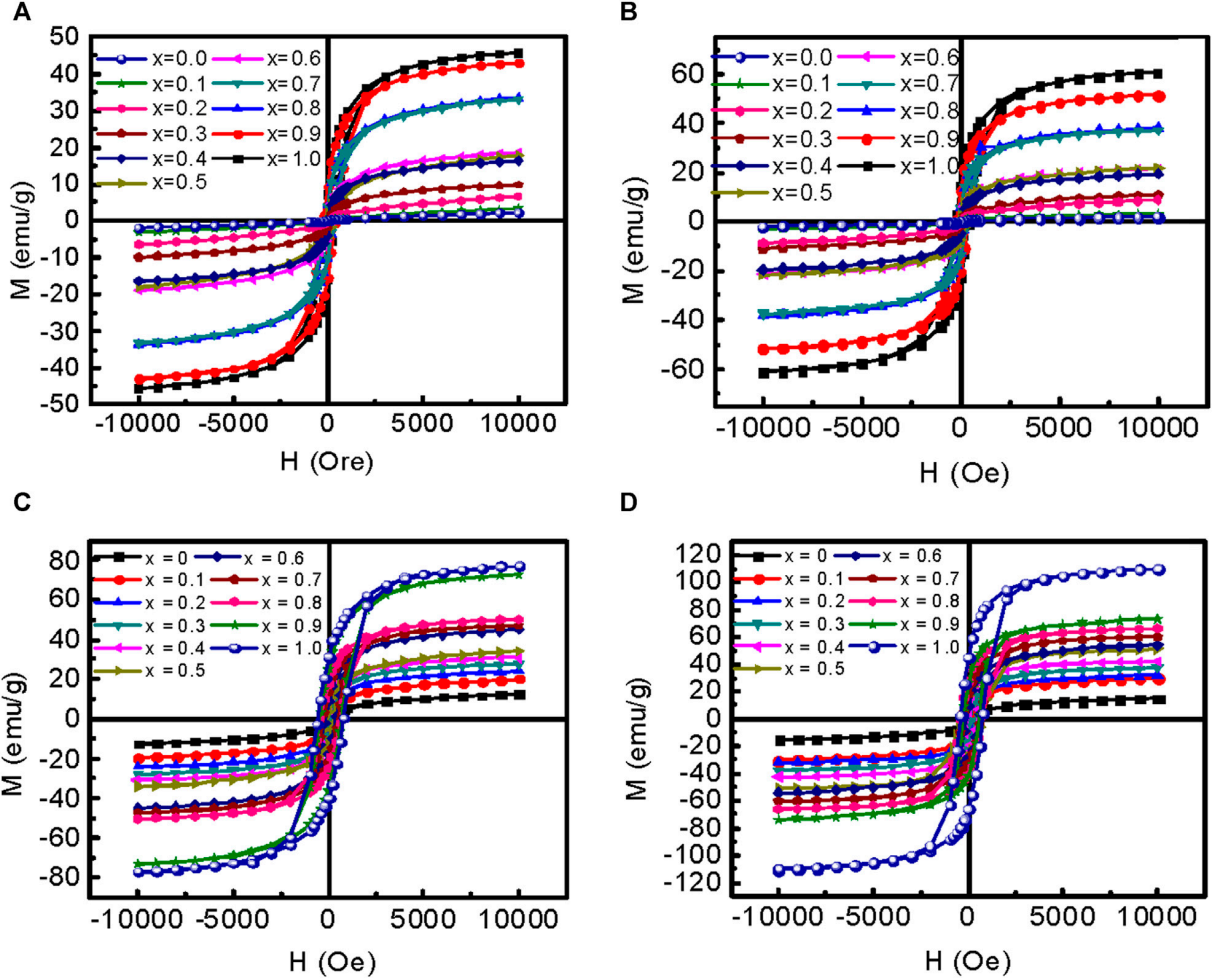
Variation of magnetization with an applied magnetic field of 10 kOe of Mg_1-*x*_Co_*x*_Fe_2_O_4_ nanoparticle (0 *≤ x ≤* 1; ∆*x* = 0.1) annealed at **(A)** 200°C, **(B)** 400°C, **(C)** 600°C, and **(D)** 800°C are presented.

**FIGURE 14:**
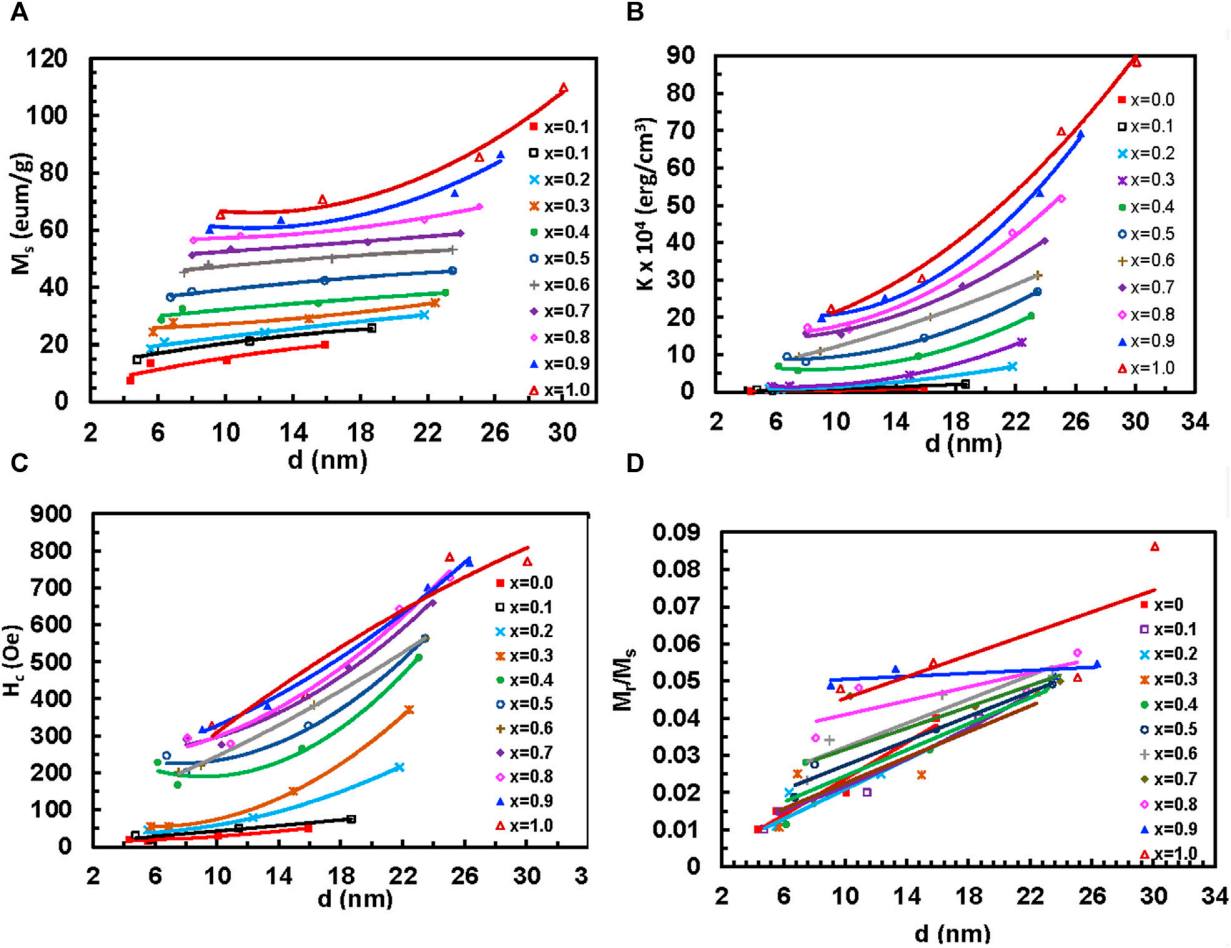
Variation of the **(A)** Coercive field, H_*c*_, **(B)** saturation magnetization, M_*s*_, **(C)** remnant ratio, M_*r*_/M_*s*_, and **(D)** anisotropy constant, K with cobalt content *x* of Mg_1-*x*_Co_*x*_Fe_2_O_4_ nanoparticle (0 *≤ x ≤* 1; ∆*x* = 0.1) annealed at **(A)** 200°C, **(B)** 400°C, **(C)** 600°C, and **(D)** 800°C.

The variation of magnetization (M) with an applied magnetic field (H) of MCFO ferrite nanoparticles annealed at 200°C–800°C are presented in Figure 13. The magnetization increases with an increase in Co^2+^ content because Co^2+^ replaces Mg^2+^, and the magnetic moment of Co^2+^ (3.88*µ*_*B*_) is higher than Mg^2+^ (0*µ*_*B*_) (Mund and Ahuja 2016; Anis-Ur-Rehman et al., 2011). The magnetization also increases with an increase in annealing temperature because of the increase in the ordered core replacing disordered surface atoms. The saturation magnetizations (M_*s*_) were determined by using Law of Approach to Saturation Jr. and Silva (2011). The anisotropy constants (K) were obtained by using the relation

$$K=\frac{H_{c}\times M_{s}}{0.96}$$ (5)

where H_*c*_ is the coercive field (Yadav et al., 2017). Figure 14 shows the variation of (M_*s*_), anisotropy constant (K), coercivity (H_c_), and remanence ratio (M_*r*_/M_*s*_) with particle size of MCFO ferrite nanoparticles with compositions and annealing temperatures. The data are presented in Supplementary Table S10. The values of M_*s*_ and M_*r*_/M_*s*_ increase with the particle size because of the increase of ferrimagnetic core at the expense of the disordered surface. Further, we observed from Raman and FTIR spectroscopy that B-site occupancy of Fe tends to increase rather than the A-site occupancy, which increases the A-B exchange interaction. The values of K and H_*c*_ increase because, with the increase of particle size, the particles become multidomain and there is an increased amount of Co. However, with the increase of Co^2+^, all the values of M_*s*_, M_*r*_/M_*s*_, K, and H_*c*_ increase because of the higher magnetic moment of Co^2+^ (3.88*µ*_*B*_) than Mg^2+^ (0*µ*_*B*_). The values of K and H_*c*_ increase with an increase in Co^2+^ content because of the higher anisotropy of CFO (Mund and Ahuja, 2016).

#### 3.1.6 Hyperthermia properties

**FIGURE 15:**
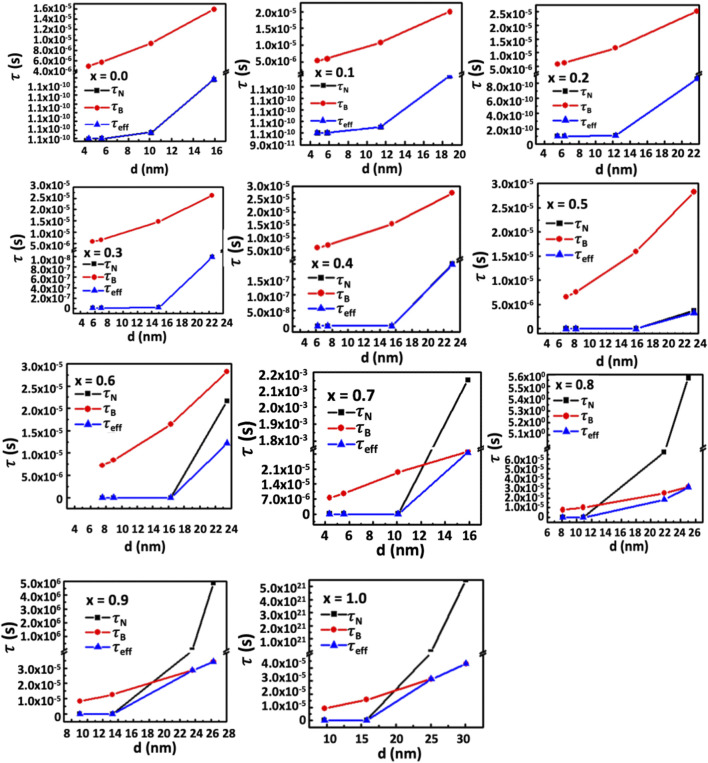
**(A–K)** The temperature evolution with time of chitosan-MCFO nanohybrids (Mg_1-*x*_Co_*x*_Fe_2_O_4_ nanoparticle; 0 *≤ x ≤* 1; ∆*x* = 0.1). The heating profiles show the evolution of temperature with particle size for the concentration of 4 mg/mL. The hyperthermia set-up consists of a sample coil of eight turns that has a 4 cm diameter. The rf magnetic field amplitude was 26 mT and the frequency 343 kHz. In each test, 600 *µ*L of nanohybrid suspension of different concentrations were transferred in an Eppendorf tube and placed inside the sample coil. The temperature was measured with a digital thermometer immediately after switching off the power supply in each case.

Figure 15 shows the time-dependence temperature evolution of chitosan-MCFO nanohybrids with different particle sizes for the entire range of composition for the concentration of 4 mg/mL. Similar experiments were also carried out for three more concentrations of 0.5, 1, and 2 mg/mL, which show similar heating profile. The temperature increases with time linearly first and then it reaches a plateau value. The temperature evolution of chitosan-MCFO nanohybrid increase with the particle size for all compositions. The temperature at the plateau region also increases with the Co^2+^ content because of the increase in magnetic moment with Co^2+^. Habib et al. (2008) reported similar behavior for Fe-Co alloy, magnetite, and maghemite nanoparticles.

**FIGURE 16:**
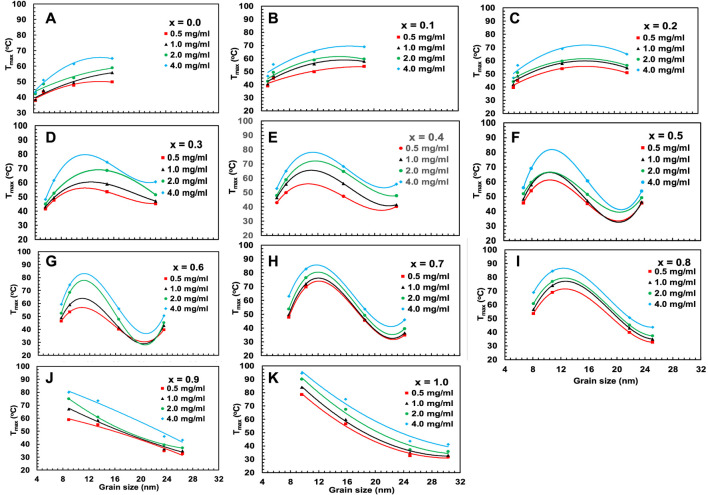
**(A–K)** Variation of maximum temperature T_max_ acquired from Figure 15 for chitosan-MCFO nanohybrids (Mg_1-*x*_Co_*x*_Fe_2_O_4_ nanoparticle; 0 *≤ x ≤* 1; ∆*x* = 0.1) of different concentrations of 0.5, 1, 2, and 4 mg/mL.

**FIGURE 17:**
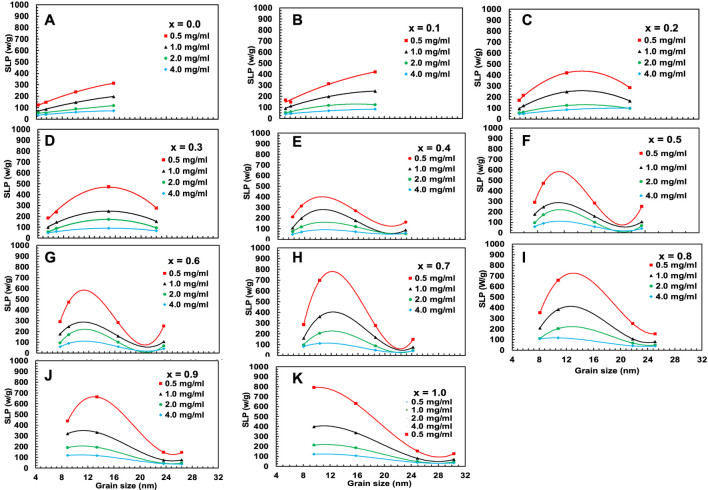
**(A–K)** Variation of specific loss power, SLP, acquired from Figure 15 for chitosan-MCFO nanohybrids (Mg_1-*x*_Co_*x*_Fe_2_O_4_ nanoparticle; 0 *≤ x ≤* 1; ∆*x* = 0.1) of different concentrations of 0.5, 1, 2, and 4 mg/mL.

Figures 16, 17 represent the variation of T_*max*_ and specific loss power (SLP) with the particle size of chitosan-MCFO nanohybrid of different concentrations. The data are presented in Supplementary Table S11, S12. Initially, the value of T_*max*_ and SLP increases with particle size for each sample, reaches a maximum, and then decreases. It is intriguing to note that both T_*max*_ and SLP exhibit similar behaviors with x and particle size. However, with the increase of nanoparticle concentration in water from 0.5 to 4 mg/mL, specific loss power decreases while T_*max*_ increases. This might be because, with the increase of concentration, Brownian relaxation will be impaired because of agglomeration of the particles at the initial stage of particle heating. This is because the initial slope of time-dependence temperature curve determines SLP. With time, when the particles get sufficient energy, this hindrance of Brownian relaxation is overcome. By the time the temperature reaches the plateau value, particles gain sufficient energy to increase T_*max*_ with concentration.

It will be interesting to revisit the expressions responsible for the self-heating properties of the nanoparticles and the dependence on the particle size and magnetic anisotropy with an alternating magnetic field. The self-heating properties of the nanoparticles are affected by the Néel and Brownian relaxations and hysteresis loss (Torres et al., 2019; Barati et al., 2014; Reeves and Weaver, 2014; Ferguson et al., 2013). By definition, specific loss power is the energy absorbed by the nanoparticles per unit volume and time and can be expressed as,

$$SLP\;\left(W/g\right)=P/\rho$$ (6)

Where,

$$P=\pi {µ}_{o}\chi_{o}H^{2}f\;\left\{2\pi f\tau_{eff}\;/\left(1+{\left(2\pi f\tau_{eff}\right)}^{2}\right)\right\}$$ (7)

P is the mean volumetric dissipation power, *ρ* is the density of the magnetic material, *χ*_*o*_ is the initial magnetic susceptibility, H is the magnetic field amplitude, *f* is the frequency, and *µ*_*o*_ is the permeability of the vacuum (Ota and Takemura, 2019). The *τ*_*eff*_ is the weighted average of the Néel and Brownian relaxations and related to the Néel and Brownian relaxations by the following formula

$$\tau_{eff}=\left(\tau_{N}+\tau_{B}\right)/\tau_{N}\;\tau_{B}$$ (8)

The Néel and Brownian relaxations are very effective for the self-heating properties of nanoparticles up to small anisotropy and particle size, which can be expressed as,

$$\tau_{N}=\tau_{o}\exp\left(KV/k_{B}T\;\right)$$ (9)

and

$$\tau_{B}=4\pi \eta r_{H}^{3}/k_{B}T$$ (10)

where *K* is the anisotropy of the magnetic nanoparticle, *V* is the volume of the magnetic core, *η* is the viscosity of the solvent, *r*_*H*_ is the hydrodynamic radius of the magnetic nanoparticle, *k*_*B*_ is the Boltzmann’s constant, and *T* is the temperature. The above equations show that the Néel relaxation is directly proportional to the anisotropy and the particle size, while the Brownian relaxation is directly proportional to the hydrodynamic diameter, which is again related to the particle size. There exists a critical diameter above which hysteresis loss comes into play for the self-heating properties of the nanoparticles (Barati et al., 2014; Islam et al., 2020),

The critical diameter can be expressed using the following relation:

$$D_{cr}={\left[6\ln\left(t_{m}f_{o}\right)k_{B}T/\pi K\right]}^{1/3}$$ (11)

**FIGURE 18:**
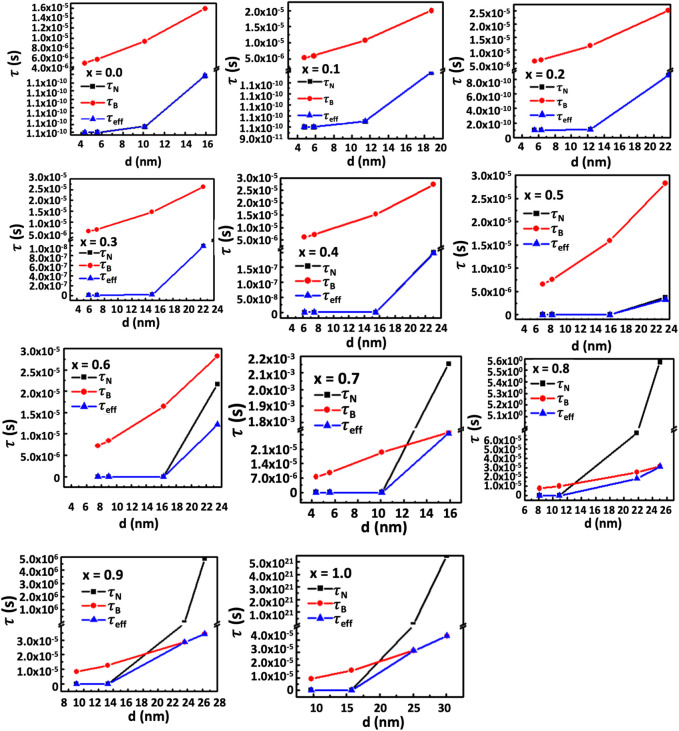
**(A–K)** The contribution of Néel relaxation time, *τ*_*N*_, Brownian relaxation time, *τ*_*B*,_ to the effective relaxation time, *τ*_*eff*_, of chitosan-MCFO nanohybrids (Mg_1-*x*_Co_*x*_Fe_2_O_4_ nanoparticle; 0 *≤ x ≤* 1; ∆*x* = 0.1) with the particle size. It is interesting to note that up to x = 0.5, *τ*_*eff*_ coincides with *τ*_*N*_ and is faster in this range of composition and particle size than *τ*_*B*_ because of the limited anisotropy. For x > 0.6, there exists a crossover below which *τ*_*eff*_ coincides with *τ*_*N*_. The *τ*_*N*_ is faster than *τ*_*B*_ in this range of particle size. Above the crossover, the *τ*_*B*_ is faster than *τ*_*N*_ and, therefore, *τ*_*eff*_ coincides with *τ*_*B*_.

Where, *K* is the anisotropy constant of MCFO, *f*_*o*_ is the frequency, *k*_*B*_ the Boltzmann’s constant, *T* the temperature, and *t*_*m*_ the measurement time (Barati et al., 2014). The given expression shows an inverse relationship between magnetic anisotropy and critical diameter. The anisotropy constant causes the critical diameter to decrease as the Co^2+^ concentration increases. Beyond the critical diameter, anisotropy exceeds the threshold for Néel relaxation. For lower Co^2+^ content and smaller particle sizes, Néel relaxation dominates the effective relaxation time, τ_eff_. However, Brownian relaxation dominates the relaxation mechanism beyond the critical diameter. Figure 18 depicts the particle size dependence of Néel and Brownian relaxation time, as well as the effective relaxation time. It is observed in the figure that the Néel relaxation, *τ*_*N*,_ is faster while Brownian relaxation, *τ*_*B*,_ is slower. Up to *x* = 0.5, the Néel relaxation time, *τ*_*N*_, and the effective relaxation time, *τ*_*eff*_, coincide fully. For *x* = 0.6, *τ*_*N*_ and *τ*_*eff*_ starts separating beyond 16 nm particle size. For *x* = 0.7 and above there is crossover at a certain particle diameter below which *τ*_*N*_ is faster and in this range *τ*_*eff*_ coincides with *τ*_*N*_. Above the crossover, *τ*_*B*_ is faster and in this range *τ*_*eff*_ coincides with *τ*_*B*_. The magnetic domain’s influence on anisotropy is widely recognized. Particles are monocrystalline and monodomain, having minimal anisotropy at small sizes. However, as particle size grows, they become multidomain, resulting in the presence of anisotropy. Anisotropy rises with particle size. As the Co^2+^ content increases, particle size and critical diameters reduce further because of the intrinsic increase of anisotropy. In Figures 16, 17, the SLP and T_max_ increase up to the critical diameter, reach a maximum, and then decrease with particle size. The foregoing rationale illustrates how anisotropy and average particle size affect these parameters.

The range of T_max_ and SLP obtained in this study can be efficiently used for the annihilation of cancer cells as we observed in our previous studies (Hoque et al., 2016; Hoque et al., 2021; Hyder and Hoque., 2017). We observed above 98% annihilation of 9L Gliosarcoma cancer cells by using CoFe_2_O_4_ and Fe_x_Co_1-x_Fe_2_O_4_ and chitosan nanohybrid as the thermotherapeutic agent for 15 min exposure to an rf magnetic field. We expect similar annihilation of cancer cells by the chitosan-MCFO nanohybrids for comparable specific loss power and T_max_.

## 4 Conclusion

The lattice parameter, the particle size, the X-ray density, the ionic radius of the tetrahedral and octahedral site, the hopping length of the tetrahedral and octahedral site, the bond length of the octahedral and tetrahedral site, cation-cation distance, and cation-anion distance of the MCFO nanoparticles increases with an increase in Co^2+^ content and annealing temperature. The TEM, HRTEM, and SAED patterns clearly demonstrate grain growth and increased crystallinity with the Co^2+^ content and annealing temperature. The FTIR and Raman spectra demonstrates strong dependence of cation distribution with x, with the spinel becoming increasingly inverse with the increase of Co^2+^. The above structural transformation causes A-B exchange interaction to be stronger, facilitating an increase in the saturation magnetization. With the increase of Co^2+^ content, x, and particle size, anisotropy also increases. A limited increase of anisotropy and particle diameter help increase Néel and Brownian relaxations, facilitating effective relaxation, which increases the efficiency of hyperthermia. Beyond a critical diameter where anisotropy exceeds the threshold for Néel relaxation, the effective relaxation is dominated by the Brownian relaxation.

## Data Availability

The original contributions presented in the study are included in the article/Supplementary Material, further inquiries can be directed to the corresponding author.

## References

[B1] Abenojar E. C. Wickramasinghe S. Bas-Concepcion J. Samia A. C. S. (2016). Structural effects on the magnetic hyperthermia properties of iron oxide nanoparticles. Prog. Nat. Sci. Mater. Int. 26, 440–448. 10.1016/j.pnsc.2016.09.004

[B2] Abraham A. G. Manikandan A. Manikandan E. Vadivel S. Jaganathan S. K. Baykal A. (2018). Enhanced magneto-optical and photo-catalytic properties of transition metal cobalt (Co^2+^ions) doped spinel MgFe_2_O_4_ ferrite nanocomposites. J. Magn. Magn. Mat. 452, 380–388. 10.1016/j.jmmm.2018.01.001

[B3] Alawi A. M. A. Majoni S. W. Falhammar H. (2018). Magnesium and human health: perspectives and research directions. Int. J. Endocrinol. 2018, 9041694. 10.1155/2018/9041694 29849626 PMC5926493

[B4] Anis-Ur-Rehman M. Malik M. A. Khan M. Maqsood K. (2011). Structural, electrical and magnetic properties of nanocrystalline Mg-Co ferrites prepared by Co-precipitation. J. Nano Res. 14, 1–9. 10.4028/www.scientific.net/jnanor.14.1

[B5] Anjum S. Tufail R. Rashid K. Zia R. Riaz S. (2017). Effect of cobalt doping on crystallinity, stability, magnetic and optical properties of magnetic iron oxide nano-particles. J. Magnetism Magnetic Mater. 432, 198–207. 10.1016/j.jmmm.2017.02.006

[B6] Baig N. Kammakakam I. Falath W. (2021). Nanomaterials: a review of synthesis methods, properties, recent progress, and challenges. Mat. Adv. 2, 1821–1871. 10.1039/d0ma00807a

[B7] Bao Y. Wen T. Samia A. C. Khandhar A. Krishnan K. M. (2015). Magnetic nanoparticles: material engineering and emerging applications in lithography and biomedicine. J. Mat. Sci. 51, 513–553. 10.1007/s10853-015-9324-2 PMC4646229 26586919

[B8] Barati M. R. Selomulya C. Suzuki K. (2014). Particle size dependence of heating power in MgFe_2_O_4_ nanoparticles for hyperthermia therapy application. J. Appl. Phys. 115. 10.1063/1.4867751

[B9] Chen R. J. Lee V. R. (2023). Cobalt toxicity. 36508548

[B10] Chintala J. N. P. K. Kaushik S. D. Varma M. C. Choudary G. S. V. R. K. Rao K. H. (2021). An accurate low temperature cation distribution of nano ni-zn ferrite having a very high saturation magnetization. J. Supercond. Nov. Magn. 34, 149–156. 10.1007/s10948-020-05728-3

[B11] Cullity B. D. (2014). Elements of X-ray diffraction. United States of America: Addison-Wesley Publishing Company Inc.

[B12] Darwish M. S. A. Kim H. Lee H. Ryu C. Lee J. Y. Yoon J. (2019). Synthesis of magnetic ferrite nanoparticles with high hyperthermia performance via a controlled co-precipitation method. J. Nanomater. (Basel) 16, 1176. 10.3390/nano9081176 PMC6724091 31426427

[B13] Denton A. R. Ashcroft N. W. (1991). Vegard’s law. Phys. Rev. A 43, 3161–3164. 10.1103/physreva.43.3161 9905387

[B14] Fang R. H. Kroll A. V. Gao W. Zhang L. (2018). Cell membrane coating nanotechnology. Adv. Mat. 30, e1706759. 10.1002/adma.201706759 PMC5984176 29582476

[B15] Ferguson R. M. Khandhar A. P. Jonasson C. Blomgren J. Johansson C. Krishnan K. M. (2013). Size-dependent relaxation properties of monodisperse magnetite nanoparticles measured over seven 465 decades of frequency by AC susceptometry. IEEE Trans. Magn. 7, 3441–3444. 10.1109/TMAG.2013.2239621 PMC4248603 25473124

[B16] Fouad D. E. Zhang C. El-Didamony H. Yingnan L. Mekuria T. D. Shah A. H. (2019). Improved size, morphology and crystallinity of hematite (*α*-Fe_2_O_3_) nanoparticles synthesized via the precipitation route using ferric sulfate precursor. Results Phys. 12, 1253–1261. 10.1016/j.rinp.2019.01.005

[B18] Gadkari A. B. Shinde T. J. Vasambekar P. N. (2009). Structural analysis of Y^3+^-doped Mg - Cd ferrites prepared by oxalate co-precipitation method. Mater. Chem. Phys. 114, 505–510. 10.1016/j.matchemphys.2008.11.011

[B19] Galinetto P. Albini B. Bini M. Mozzati M. C. (2018). “Raman spectroscopy in zinc ferrites nanoparticles,” in Raman spectroscopy. Editor do Nascimento G. M., (IntechOpen), 223–251. 10.5772/intechopen.72864

[B20] Habib A. H. Ondeck C. L. Chaudhary P. Bockstaller M. R. Mchenry M. E. (2008). Evaluation of iron-cobalt/ferrite core-shell nanoparticles for cancer thermotherapy. J. Appl. Phys. 103, 7–10. 10.1063/1.2830975

[B21] Hoque S. M. Huang Y. Cocco E. Maritim S. Santin A. D. Shapiro E. M. (2016). Improved specific loss power on cancer cells by hyperthermia and MRI contrast of hydrophilic FexCo1-xFe2O4 nanoensembles. Contrast Media Mol. Imaging 11, 514–526. 10.1002/cmmi.1713 27659164

[B22] Hoque S. M. Islam M. K. Hoq A. Haque M. M. Maritim S. Coman D. (2021). Comparative study of specific loss power and transverse relaxivity of spinel ferrite nanoensembles coated with chitosan and polyethylene glycol. Front. Nanotechnol. 3, 644080. 10.3389/fnano.2021.644080

[B23] Hyder F. Hoque S. M. (2017). Brain tumor diagnostics and therapeutics with superparamagnetic ferrite nanoparticles. Contrast Media Mol. Imaging 2017, 6387217. 10.1155/2017/6387217 29375280 PMC5742516

[B24] Islam K. Haque M. Kumar A. Hoq A. Hyder F. Hoque S. M. (2020). Manganese ferrite nanoparticles (MnFe_2_O_4_): size dependence for hyperthermia and negative/positive contrast enhancement in MRI. Nanomaterials 10, 2297. 10.3390/nano10112297 33233590 PMC7699708

[B25] Islam M. A. Hasan M. R. Haque M. M. Rashid R. Syed I. M. Hoque S. M. (2022). Efficacy of surface-functionalized Mg_1– x_Co_x_Fe_2_O_4_ (0*≤ x≤* 1; ∆ x= 0.1) for hyperthermia and *in vivo* MR imaging as a contrast agent. RSC Adv. 12, 7835–7849. 10.1039/d2ra00768a 35424744 PMC8982169

[B26] Josyulu O. S. Sobhanadri J. (1981). The far-infrared spectra of some mixed cobalt zinc and magnesium zinc ferrites. Phys. Stat. Sol. (a) 65, 479–483. 10.1002/pssa.2210650209

[B27] Kafrouni L. Savadogo O. (2016). Recent progress on magnetic nanoparticles for magnetic Hyperthermia. Prog. Biomater. 5, 147–160. 10.1007/s40204-016-0054-6 27995583 PMC5304434

[B28] Kumar G. Rani R. Sharma S. Batoo K. M. Singh M. (2013). Electric and dielectric study of cobalt substituted MgMn nanoferrites synthesized by solution combustion technique. Ceram. Int. 39, 4813–4818. 10.1016/j.ceramint.2012.11.071

[B29] Li J. Cai C. Li J. Li J. Li J. Sun T. (2018). Chitosan-Based nanomaterials for drug delivery. Molecules 23, 2661. 10.3390/molecules23102661 30332830 PMC6222903

[B30] Li X. Sun Y. Zong Y. Wei Y. Liu X. Li X. (2020). Size-effect induced cation redistribution on the magnetic properties of well-dispersed CoFe_2_O_4_ nanocrystals. J. Alloys Compd. 841, 155710. 10.1016/j.jallcom.2020.155710

[B31] Liu C. Zou B. Rondinone A. J. Zhang Z. J. (2000). Chemical control of superparamagnetic properties of magnesium and cobalt spinel ferrite nanoparticles through atomic level magnetic couplings. J. Am. Chem. Soc. 122, 6263–6267. 10.1021/ja000784g

[B32] Liu X. Zhang Y. Wang Y. Zhu W. Li G. Ma X. (2020). Comprehensive understanding of magnetic hyperthermia for improving antitumor therapeutic efficacy. Theranostics 10, 3793–3815. 10.7150/thno.40805 32206123 PMC7069093

[B33] Martinez-Boubeta C. Simeonidis K. Makridis A. Angelakeris M. Iglesias O. Guardia P. (2013). Learning from nature to improve the heat generation of iron-oxide nanoparticles for magnetic hyperthermia applications. Sci. Rep. 3, 1652. 10.1038/srep01652 23576006 PMC3622918

[B34] Mcbain S. C. Yiu H. H. Dobson J. (2008). Magnetic nanoparticles for gene and drug delivery. Int. J. Nanomedicine 3, 169–180. 10.2147/ijn.s1608 18686777 PMC2527670

[B35] Mohapatra J. Xing M. Liu J. P. (2019). Inductive thermal effect of ferrite magnetic nanoparticles. Materials 12, 3208. 10.3390/ma12193208 31574950 PMC6804282

[B36] Mund H. S. Ahuja B. L. (2016). Structural and magnetic properties of Mg doped cobalt ferrite nanoparticles prepared by sol-gel method. Mat. Res. Bull. 85, 228–233. 10.1016/j.materresbull.2016.09.027

[B37] Naseri M. G. Ara M. H. M. Saion E. B. Shaari A. H. (2014). Superparamagnetic magnesium ferrite nanoparticles fabricated by a simple, thermal-treatment method. J. Magn. Magn. Mat. 350, 141–147. 10.1016/j.jmmm.2013.08.032

[B38] Nemala H. Thakur J. S. Naik V. M. Vaishnava P. P. Lawes G. Naik R. (2015). Investigation of temperature dependent magnetic hyperthermia in Fe_3_O_4_ ferrofluids. J. Appl. Phys. 116, 034309. 10.1063/1.4890456

[B39] Nlebedim I. C. Ranvah N. Williams P. I. Melikhov Y. Snyder J. E. Moses A. J. (2010). Effect of heat treatment on the magnetic and magnetoelastic properties of cobalt ferrite. J. Magn. Magn. Mat. 322, 1929–1933. 10.1016/j.jmmm.2010.01.009

[B40] Ota S. Takemura Y. (2019). Characterization of Néel and Brownian relaxations isolated from complex dynamics influenced by dipole interactions in magnetic nanoparticles. J. Phys. Chem. C 123, 28859–28866. 10.1021/acs.jpcc.9b06790

[B41] Pawlak A. Mucha M. (2003). Thermogravimetric and FTIR studies of chitosan blends. Thermochim. Acta 396, 153–166. 10.1016/s0040-6031(02)00523-3

[B42] Rajan A. Sahu N. K. (2020). Review on magnetic nanoparticle-mediated Hyperthermia for cancer therapy. J. Nanoparticle Res. 22, 319. 10.1007/s11051-020-05045-9

[B43] Rana S. Philip J. Raj B. (2010). Micelle based synthesis of cobalt ferrite nanoparticles and its characterization using Fourier Transform Infrared Transmission Spectrometry and Thermogravimetry. Mat. Chem. Phys. 124, 264–269. 10.1016/j.matchemphys.2010.06.029

[B44] Reeves D. B. Weaver J. B. (2014). Approaches for modeling magnetic nanoparticle dynamics. Crit. Rev. Biomed. Eng. 42, 85–93. 10.1615/critrevbiomedeng.2014010845 25271360 PMC4183932

[B45] Rhee S. Hong S. Chang Y. (2010). Chitosan-coated ferrite (Fe3O4) nanoparticles as a T2 contrast agent for magnetic resonance imaging. J. Korean Phys. Soc. 56, 868–873. 10.3938/jkps.56.868

[B46] Satalkar M. Kane S. N. (2016). On the study of Structural properties and Cation distribution of Zn_0.75-x_Ni_x_Mg_0.15_Cu_0.1_Fe_2_O_4_ nano ferrite: effect of Ni addition. J. Phys. Conf. Ser. 755, 012050. 10.1088/1742-6596/755/1/012050

[B47] Sensenig R. Sapir Y. MacDonald C. Cohen S. Polyak B. (2012). Magnetic nanoparticle-based approaches to locally target therapy and enhance tissue regeneration *in vivo* . Nanomedicine (Lond) 7, 1425–1442. 10.2217/nnm.12.109 22994959 PMC3543693

[B48] Sensenig R. Sapir Y. MacDonald C. Cohen S. Polyak B. (2014). Heating efficiency in magnetic nanoparticle hyperthermia. J. Magn. Magn. Mat. 354, 163–172. 10.1016/j.jmmm.2013.11.006

[B49] Sharma R. Thakur P. Kumar M. Thakur N. Negi N. S. Sharma P. (2016). Improvement in magnetic behaviour of cobalt doped magnesium zinc nano-ferrites via co-precipitation route. J. Alloys Compd. 684, 569–581. 10.1016/j.jallcom.2016.05.200

[B50] Soler M. A. G. Melo T. F. O. da Silva S. W. Lima E. C. D. Pimenta A. C. M. Garg V. K. (2004). Structural stability study of cobalt ferrite-based nanoparticle using micro Raman spectroscopy. J. Magn. Magn. Mat. 272–276, 2357–2358. 10.1016/j.jmmm.2003.12.582

[B51] Tatarchuk T. Bououdina M. Vijaya J. J. Kennedy L. J. (2017). “Spinel ferrite nanoparticles: synthesis, crystal structure, properties, and perspective applications,” in Nanophysics, nanomaterials, interface studies, and applications. NANO 2016. Springer proceedings in Physics, Fesenko O. Yatsenko L., (Cham: Springer), 195. 10.1007/978-3-319-56422-7_22

[B52] Torres T. E. Lima E. Calatayud M. P. Sanz B. Ibarra A. Fernández-Pacheco R. (2019). The relevance of Brownian relaxation as power absorption mechanism in Magnetic Hyperthermia. Sci. Rep. 9, 3992. 10.1038/s41598-019-40341-y 30850704 PMC6408542

[B53] Tran N. Webster T. J. (2010). Magnetic nanoparticles: biomedical applications and challenges. J. Mat. Chem. 20, 8760–8767. 10.1039/c0jm00994f

[B54] Valério A. Morelhao S. L. (2019). Usage of Scherrer’s formula in X-ray diffraction analysis of size distribution in systems of monocrystalline nanoparticles. *arXiv* , arXiv:1911.00701.

[B55] Vijaya M. L. K. Thyagarajan K. K. (2015). An investigation of structural and magnetic properties of Cr - Zn ferrite nanoparticles prepared by a sol - gel process. J. Nanostructure Chem. 5, 365–373. 10.1007/s40097-015-0168-8

[B56] Yadav R. S. Kurtka I. Vilcakova J. Havlica J. Masilko J. Kalina L. (2017). Impact of grain size and structural changes on magnetic, dielectric, electrical, impedance and modulus spectroscopic characteristics of CoFe_2_O_4_ nanoparticles synthesized by honey mediated sol-gel combustion method. Adv. Nat. Sci. Nanosci. Nanotechnol. 8, 045002. 10.1088/2043-6254/aa853a

[B57] Zeeshan T. Anjum S. Iqbal H. Zia R. (2018). Substitutional effect of copper on the cation distribution in cobalt chromium ferrites and their structural and magnetic properties. Mater. Pol. 36, 255–263. 10.1515/msp-2018-0011

