# Supplementary material for: Effect of particle size and composition on local magnetic hyperthermia of chitosan-Mg1−xCoxFe2O4 nanohybrid
Source: Front Chem. 2024 Mar 7;12:1347423. doi: 10.3389/fchem.2024.1347423 (PMC10958782; doi:10.3389/fchem.2024.1347423)
Supplement: Supplementary file 1 [file DataSheet1.PDF]

Supplementary Material

X-ray Diffraction

The particle sizes were calculated using Debye-Scherrer's formula:

$$D = \frac{0.94\lambda}{\beta cos\theta} \quad (1)$$

The observed lattice parameter for each plane was calculated using Bragg's law for the cubic structure which is written as

$$d_{hkl} = \frac{a}{\sqrt{h^2+k^2+l^2}} \quad (2)$$

where h, k, l are Miller indices[25]. The precise values of the lattice parameters were determined using the Nelson-Riley function

$$F(\theta) = \frac{1}{2}\left(\frac{cos^2\theta}{sin\theta} + \frac{cos^2\theta}{\theta}\right) \quad (3)$$

The values of lattice constant, a for each plane are plotted as a function of $F(\theta)$ and the true value of the lattice parameter was found from the intercept of the linear extrapolation of a_0 vs. $F(\theta)$ lines[26,27].

The X-ray density (d_x) of spinel ferrite nanoparticles can be written as

$$d_x = \frac{8M}{N_0 a^3} \quad (4)$$

where M is the molecular weight of the sample, N_o is Avogadro's number (6.0225 × 1023 particles/mole), and a is the lattice parameter[31].

The specific surface area of the particles (S), the radius of the tetrahedral site (r_A), the radius of the octahedral site (r_B), the theoretical lattice parameter (a_{th}), the hopping length for the tetrahedral site (d_A), hopping length for the octahedral site (d_B), the tetrahedral and the octahedral bond length ($d_{A\times}$ and $d_{B\times}$), the tetrahedral edge ($d_{A\times E}$), the shared and unshared octahedral edge ($d_{B\times E}$ and $d_{B\times EU}$) for cubic spinel ferrite nanoparticles can be written as

$$S = \frac{6}{D \times d_x} \quad (5)$$

$$r_A = (u - 0.25)a\sqrt{3} - R_0 \quad (6)$$

$$r_B = (0.625 - u)a - R_0 \quad (7)$$

$$a_{th} = \frac{8}{3\sqrt{3}}[(r_A - R_0) + (r_B - R_0)] \quad (8)$$

$$d_A = 0.25a\sqrt{3} \quad (9)$$

$$d_B = 0.25a\sqrt{2} \quad (10)$$

$$d_{AX} = a\sqrt{3}\left(u - \frac{1}{4}\right) \quad (11)$$

$$d_{BX} = a\left[3u^2 - \left(\frac{11}{4}\right)u + \left(\frac{43}{64}\right)\right]^{\frac{1}{2}} \quad (12)$$

$$d_{AXE} = a\sqrt{2}\left(2u - \frac{1}{2}\right) \quad (13)$$

$$d_{BXE} = a\sqrt{2}(1 - 2u) \quad (14)$$

$$d_{BXEU} = a\left[4u^2 - 3u + \left(\frac{11}{16}\right)\right]^{\frac{1}{2}} \quad (15)$$

where u (0.381) is the oxygen positional parameter, a is the lattice constant, and R_0 (1.32 Å) the oxygen ion radius[31–33]. Table S1-S4 presents the variation of S, r_A, r_B, d_A, d_B, d_{AX}, d_{BX}, d_{AXE}, d_{BXE}, and d_{BXEU} with Co^{2+} content of $Mg_{1-x}Co_xFe_2O_4$ ($0 \leq x \leq 1$ with $\Delta x = 0.1$) nanoparticles annealed at 200°C, 400°C, 600°C, and 800°C.The value of S decreases with an increase in Co^{2+} content and annealing temperature because particle size increases with an increase in Co^{2+} content and annealing temperature. The value of r_A, r_B, d_A, d_B, d_{AX}, d_{BX},d_{AXE}, d_{BXE}, and d_{BXEU} increases with an increase in Co^{2+} content and annealing temperature because particle size increases with an increase in Co^{2+} content and annealing temperature which is associated with cation redistribution on grain size growth.

The interionic distances between cations $b, c, d, e,$ and f were obtained by the equations which are written as

$$b = (a\ /\ 4)\sqrt{2} \quad (16)$$
$$c = (a/8)\sqrt{11} \quad (17)$$
$$d = (a/4)\sqrt{3} \quad (18)$$
$$e = (3a/8)\sqrt{3} \quad (19)$$
$$f = (a/4)\sqrt{3} \quad (20)$$

The distances between cations and anions $p,\ q,\ r,$ and s were found by using the relations

$$p = a\left(1/2 - u^{\bar{3}m}\right) \quad (21)$$
$$q = a\left(u^{\bar{3}m} - 1/8\right)\sqrt{3} \quad (22)$$
$$r = a\left(u^{\bar{3}m} - 1/8\right)\sqrt{11} \quad (23)$$
$$s = a_{exp}/3\left(u^{\bar{3}m} - 1/2\right)\sqrt{3} \quad (24)$$

The bond angles were calculated by using the relations

$$\theta_1 = cos^{-1}((p^2 + q^2 - c^2)/2pq) \quad (25)$$
$$\theta_2 = cos^{-1}((p^2 + r^2 - e^2)/2pr) \quad (26)$$
$$\theta_3 = cos^{-1}((2p^2 - b^2)/2pr) \quad (27)$$
$$\theta_4 = cos^{-1}((p^2 + s^2 - f^2)/2ps) \quad (28)$$
$$\theta_5 = cos^{-1}((r^2 + q^2 - d^2)/2rq) \quad (29)$$

Table S5-S8 present the cation-cation distances $b, c, d, e,$ and f and the cation-anion distances $p, q, r,$ and s and the bond angles θ_1, θ_2, θ_3, θ_4, and θ_5 with cobalt content x. The cation-cation distances and cation-anion distances increase with an increase in Co^{2+} content and annealing temperature because the ionic radius of Co^{2+}(0.072 nm) is higher than that of Mg^{2+}(0.065 nm).

Table S1. Variation of the grain size D, the observed lattice parameter a, the X-ray density, the specific surface area of the particles S, the ionic radius of the tetrahedral site r_A, the ionic radius of octahedral site r_B, the theoretical lattice parameter a_{th}, the hopping length for tetrahedral site d_A, the hopping length for octahedral site d_B, the bond length of tetrahedral site d_{AX}, the bond length of octahedral site d_{BX}, the tetrahedral edge d_{AXE}, the shared octahedral edge d_{BXE}, and the unshared octahedral edge d_{BXEU} of MCFO nanoparticles at different values of x and annealed at 200°C.

x	g.s.	a	d_x	S	r_A	r_B	a_{th}	d_A	d_B	d_{AX}	d_{BX}	d_{AXE}	d_{BXE}	d_{BXEU}
0	3.8	8.33	4.59584	3.04E+06	0.57007	0.71252	8.33216	3.607	2.9451	1.89007	2.03375	3.08646	2.80373	2.9468
0.1	4.0	8.341	4.65694	2.63E+06	0.57256	0.7152	8.34316	3.61176	2.94899	1.89256	2.03643	3.09054	2.80744	2.95069
0.2	4.5	8.339	4.73961	2.30E+06	0.57211	0.71472	8.34116	3.61089	2.94828	1.89211	2.03595	3.0898	2.80676	2.94998

0.3	4.5	8.345	4.80651	2.23E+06	0.57347	0.71618	8.34716	3.61349	2.9504	1.89347	2.03741	3.09202	2.80878	2.9521
0.4	5.2	8.349	4.88066	1.98E+06	0.57438	0.71716	8.35117	3.61522	2.95182	1.89438	2.03839	3.0935	2.81013	2.95352
0.5	5.8	8.352	4.95435	1.78E+06	0.57506	0.71789	8.35417	3.61652	2.95288	1.89506	2.03912	3.09462	2.81114	2.95458
0.6	6.6	8.359	5.02066	1.57E+06	0.57665	0.7196	8.36117	3.61955	2.95535	1.89665	2.04083	3.09721	2.8135	2.95705
0.7	6.6	8.357	5.10307	1.49E+06	0.57619	0.71911	8.35917	3.61869	2.95465	1.89619	2.04034	3.09647	2.81282	2.95635
0.8	6.7	8.365	5.16703	1.43E+06	0.57801	0.72106	8.36717	3.62215	2.95747	1.89801	2.04229	3.09943	2.81552	2.95918
0.9	7.0	8.369	5.23809	1.29E+06	0.57891	0.72204	8.37117	3.62388	2.95889	1.89891	2.04327	3.10091	2.81686	2.96059
1	7.3	8.375	5.30514	1.18E+06	0.58028	0.7235	8.37717	3.62648	2.96101	1.90028	2.04474	3.10314	2.81888	2.96271

Table S2. Variation of the grain size D, the observed lattice parameter a, the X-ray density, the specific surface area of the particles S, the ionic radius of the tetrahedral site r_A, the ionic radius of octahedral site r_B, the theoretical lattice parameter a_{th}, the hopping length for tetrahedral site d_A, the hopping length for octahedral site d_B, the bond length of tetrahedral site d_{AX}, the bond length of octahedral site d_{BX}, the tetrahedral edge d_{AXE}, the shared octahedral edge d_{BXE}, and the unshared octahedral edge d_{BXEU} of MCFO nanoparticles at different values of x and annealed at 400°C.

x	g.s.	a	d_x	S	r_A	r_B	a_{th}	d_A	d_B	d_{AX}	d_{BX}	d_{AXE}	d_{BXE}	d_{BXEU}
0	5.5	8.343	4.57439	2.38E+06	0.57302	0.71569	8.34516	3.61262	2.9497	1.89302	2.03692	3.09128	2.80811	2.95139
0.1	5.7	8.35	4.6419	2.27E+06	0.5746	0.7174	8.35217	3.61566	2.95217	1.8946	2.03863	3.09388	2.81047	2.95387
0.2	6.1	8.355	4.71243	2.09E+06	0.57574	0.71862	8.35717	3.61782	2.95394	1.89574	2.03985	3.09573	2.81215	2.95564
0.3	6.9	8.353	4.79271	1.81E+06	0.57528	0.71813	8.35517	3.61696	2.95323	1.89528	2.03936	3.09499	2.81148	2.95493
0.4	7.5	8.36	4.86142	1.65E+06	0.57687	0.71984	8.36217	3.61999	2.95571	1.89687	2.04107	3.09758	2.81383	2.95741
0.5	7.9	8.365	4.93129	1.54E+06	0.57801	0.72106	8.36717	3.62215	2.95747	1.89801	2.04229	3.09943	2.81552	2.95918
0.6	8.9	8.371	4.9991	1.35E+06	0.57937	0.72252	8.37317	3.62475	2.9596	1.89937	2.04376	3.10166	2.81753	2.9613
0.7	10.3	8.37	5.07933	1.15E+06	0.57914	0.72228	8.37217	3.62432	2.95924	1.89914	2.04351	3.10129	2.8172	2.96095
0.8	10.8	8.375	5.14854	1.08E+06	0.58028	0.7235	8.37717	3.62648	2.96101	1.90028	2.04474	3.10314	2.81888	2.96271
0.9	13.4	8.379	5.21936	857885.8	0.58118	0.72448	8.38117	3.62821	2.96242	1.90118	2.04571	3.10462	2.82023	2.96413
1	15.8	8.378	5.29944	716579.2	0.58096	0.72423	8.38017	3.62778	2.96207	1.90096	2.04547	3.10425	2.81989	2.96378

Table S3. Variation of the grain size D, the observed lattice parameter a, the X-ray density, the specific surface area of the particles S, the ionic radius of the tetrahedral site r_A, the ionic radius of octahedral site r_B, the theoretical lattice parameter a_{th}, the hopping length for tetrahedral site d_A, the hopping length for octahedral site d_B, the bond length of tetrahedral site d_{AX}, the bond length of octahedral site d_{BX}, the tetrahedral edge d_{AXE}, the shared octahedral edge d_{BXE}, and the unshared octahedral edge d_{BXEU} of MCFO nanoparticles at different values of x and annealed at 600°C.

x	g.s.	a	dx	S	r_A	r_B	a_{th}	d_A	d_B	d_{AX}	d_{BX}	d_{AXE}	d_{BXE}	d_{BXEU}
0	10.1	8.368	4.53351	1.31E+06	0.57869	0.72179	8.37017	3.62345	2.95853	1.89869	2.04303	3.10054	2.81653	2.96024

0.1	11.5	8.371	4.60705	1.13E+06	0.57937	0.72252	8.37317	3.62475	2.9596	1.89937	2.04376	3.10166	2.81753	2.9613
0.2	12.5	8.375	4.67875	1.03E+06	0.58028	0.7235	8.37717	3.62648	2.96101	1.90028	2.04474	3.10314	2.81888	2.96271
0.3	15	8.378	4.74994	842116.7	0.58096	0.72423	8.38017	3.62778	2.96207	1.90096	2.04547	3.10425	2.81989	2.96378
0.4	15.6	8.381	4.82497	797135.4	0.58164	0.72496	8.38317	3.62908	2.96313	1.90164	2.0462	3.10536	2.8209	2.96484
0.5	15.9	8.383	4.89959	770183.6	0.58209	0.72545	8.38517	3.62995	2.96384	1.90209	2.04669	3.1061	2.82157	2.96554
0.6	16.5	8.385	4.9741	731059	0.58255	0.72594	8.38717	3.63081	2.96455	1.90255	2.04718	3.10684	2.82225	2.96625
0.7	18.6	8.386	5.05031	638733.9	0.58277	0.72618	8.38817	3.63124	2.9649	1.90277	2.04742	3.10721	2.82258	2.96661
0.8	21.8	8.388	5.12464	537070.7	0.58323	0.72667	8.39018	3.63211	2.96561	1.90323	2.04791	3.10795	2.82326	2.96731
0.9	23.6	8.387	5.20444	488501.2	0.583	0.72643	8.38918	3.63168	2.96525	1.903	2.04767	3.10758	2.82292	2.96696
1	24.9	8.411	5.23731	460091	0.58844	0.73228	8.41318	3.64207	2.97374	1.90844	2.05352	3.11648	2.831	2.97545

Table S4. Variation of the grain size D, the observed lattice parameter a, the X-ray density, the specific surface area of the particles S, the ionic radius of the tetrahedral site r_A, the ionic radius of octahedral site r_B, the theoretical lattice parameter a_{th}, the hopping length for tetrahedral site d_A, the hopping length for octahedral site d_B, the bond length of tetrahedral site d_{AX}, the bond length of octahedral site d_{BX}, the tetrahedral edge d_{AXE}, the shared octahedral edge d_{BXE}, and the unshared octahedral edge d_{BXEU} of MCFO nanoparticles at different values of x and annealed at 800°C.

x	g.s.	a	dx	S	r_A	r_B	a_{th}	d_A	d_B	d_{AX}	d_{BX}	d_{AXE}	d_{BXE}	d_{BXEU}
0	15.9	8.375	4.52215	834466.4	0.58028	0.7235	8.37717	3.62345	2.96101	1.90028	2.04474	3.10314	2.81888	2.96271
0.1	18.6	8.377	4.59716	701695.9	0.58073	0.72399	8.37917	3.62475	2.96172	1.90073	2.04522	3.10388	2.81955	2.96342
0.2	21.8	8.378	4.67373	588886.4	0.58096	0.72423	8.38017	3.62648	2.96207	1.90096	2.04547	3.10425	2.81989	2.96378
0.3	22.4	8.383	4.74144	564927.7	0.58209	0.72545	8.38517	3.62778	2.96384	1.90209	2.04669	3.1061	2.82157	2.96554
0.4	23.1	8.386	4.81634	539289.3	0.58277	0.72618	8.38817	3.62908	2.9649	1.90277	2.04742	3.10721	2.82258	2.96661
0.5	23.6	8.391	4.88559	520381.9	0.58391	0.7274	8.39318	3.62995	2.96667	1.90391	2.04864	3.10907	2.82427	2.96837
0.6	23.5	8.396	4.95458	515319.5	0.58504	0.72862	8.39818	3.63081	2.96843	1.90504	2.04986	3.11092	2.82595	2.97014
0.7	24.2	8.392	5.03949	491982.2	0.58413	0.72765	8.39418	3.63124	2.96702	1.90413	2.04889	3.10944	2.8246	2.96873
0.8	25.1	8.412	5.0809	470475.2	0.58867	0.73253	8.41418	3.63211	2.97409	1.90867	2.05377	3.11685	2.83133	2.9758
0.9	26.4	8.415	5.15266	441078.8	0.58935	0.73326	8.41718	3.63168	2.97515	1.90935	2.0545	3.11796	2.83234	2.97686
1	30.3	8.419	5.22239	379174.5	0.59026	0.73424	8.42118	3.64207	2.97657	1.91026	2.05548	3.11944	2.83369	2.97828

Table S5. Variation of the interionic distances between the cations b, c, d, e, f, the cation-anion distance p, q, r, s, and the bond angles θ_1, θ_2, θ_3, θ_4, θ_5, of $Mg_{1-x}Co_xFe_2O_4$ nanoparticles annealed at 200°C.

x	b	c	d	e	f	p	q	r	s	θ_1	θ_2	θ_3	θ_4	θ_5
0	2.9451	3.4534	3.6070	5.41049	5.10106	2.03252	1.89007	3.6192	3.63585	123.3401	123.3401	123.3401	125.918	74.48082
0.1	2.9489	3.458	3.61176	5.41764	5.1078	2.0352	1.89256	3.62398	3.64065	123.3406	123.3406	123.3406	125.9184	74.48074

0.2	2.9482	3.4571	3.61089	5.41634	5.10657	2.03472	1.89211	3.62311	3.63978	123.3399	123.3399	123.3399	125.9177	74.48062
0.3	2.9504	3.4596	3.61349	5.42024	5.11025	2.03618	1.89347	3.62572	3.6424	123.3397	123.3397	123.3397	125.9181	74.4806
0.4	2.9518	3.4613	3.61522	5.42283	5.1127	2.03716	1.89438	3.62746	3.64414	123.3395	123.3395	123.3395	125.9182	74.48047
0.5	2.9528	3.4625	3.61652	5.42478	5.11453	2.03789	1.89506	3.62876	3.64545	123.34	123.34	123.34	125.9179	74.48059
0.6	2.9553	3.4654	3.61955	5.42933	5.11882	2.0396	1.89665	3.6318	3.64851	123.3397	123.3397	123.3397	125.9178	74.48058
0.7	2.9546	3.4646	3.61869	5.42803	5.1176	2.03911	1.89619	3.63093	3.64764	123.3401	123.3401	123.3401	125.918	74.48085
0.8	2.9574	3.4679	3.62215	5.43323	5.1225	2.04106	1.89801	3.63441	3.65113	123.3401	123.3401	123.3401	125.9182	74.48057
0.9	2.9588	3.4696	3.62388	5.43582	5.12494	2.04204	1.89891	3.63615	3.65287	123.3398	123.3398	123.3398	125.9178	74.48051
1	2.9610	3.4720	3.62648	5.43972	5.12862	2.0435	1.90028	3.63875	3.65549	123.3397	123.3397	123.3397	125.9182	74.48069

Table S6. Variation of the interionic distances between the cations b, c, d, e, f, the cation-anion distance p, q, r, s, and the bond angles θ_1, θ_2, θ_3, θ_4, θ_5, of $Mg_{1-x}Co_xFe_2O_4$ nanoparticles annealed at 400°C.

x	b	c	d	e	f	p	q	r	s	θ_1	θ_2	θ_3	θ_4	θ_5
0	2.9497	3.45883	3.61262	5.41894	5.10902	2.03569	1.89302	3.62485	3.64153	123.3402	144.9471	92.85393	125.9178	74.48048
0.1	2.95217	3.46173	3.61566	5.42348	5.11331	2.0374	1.8946	3.62789	3.64458	123.3404	144.9466	92.85362	125.9181	74.48086
0.2	2.95394	3.4638	3.61782	5.42673	5.11637	2.03862	1.89574	3.63006	3.64676	123.3399	144.9469	92.85371	125.9181	74.48072
0.3	2.95323	3.46297	3.61696	5.42543	5.11515	2.03813	1.89528	3.62919	3.64589	123.3403	144.9471	92.85371	125.9183	74.48099
0.4	2.95571	3.46587	3.61999	5.42998	5.11943	2.03984	1.89687	3.63223	3.64895	123.34	144.9473	92.8538	125.9177	74.48098
0.5	2.95747	3.46795	3.62215	5.43323	5.1225	2.04106	1.89801	3.63441	3.65113	123.3401	144.9469	92.85348	125.9182	74.48057
0.6	2.9596	3.47043	3.62475	5.43712	5.12617	2.04252	1.89937	3.63701	3.65375	123.34	144.9471	92.85407	125.9181	74.48083
0.7	2.95924	3.47002	3.62432	5.43647	5.12556	2.04228	1.89914	3.63658	3.65331	123.3402	144.9465	92.85357	125.9182	74.48082
0.8	2.96101	3.47209	3.62648	5.43972	5.12862	2.0435	1.90028	3.63875	3.65549	123.3397	144.9469	92.85366	125.9182	74.48069
0.9	2.96242	3.47375	3.62821	5.44232	5.13107	2.04448	1.90118	3.64049	3.65724	123.34	144.9466	92.85326	125.9179	74.48063
1	2.96207	3.47334	3.62778	5.44167	5.13046	2.04423	1.90096	3.64006	3.6568	123.3402	144.9466	92.85376	125.9184	74.48054

Table S7. Variation of the interionic distances between the cations b, c, d, e, f, the cation-anion distance p, q, r, s, and the bond angles θ_1, θ_2, θ_3, θ_4, θ_5, of $Mg_{1-x}Co_xFe_2O_4$ nanoparticles annealed at 600°C.

x	b	c	d	e	f	p	q	r	s	θ_1	θ_2	θ_3	θ_4	θ_5
0	2.95853	3.46919	3.62345	5.43518	5.12433	2.04179	1.89869	3.63571	3.65244	123.34	144.9474	92.85357	125.9179	74.4807
0.1	2.9596	3.47043	3.62475	5.43712	5.12617	2.04252	1.89937	3.63701	3.65375	123.34	144.9471	92.85407	125.9181	74.48083
0.2	2.96101	3.47209	3.62648	5.43972	5.12862	2.0435	1.90028	3.63875	3.65549	123.3397	144.9469	92.85366	125.9182	74.48069
0.3	2.96207	3.47334	3.62778	5.44167	5.13046	2.04423	1.90096	3.64006	3.6568	123.3402	144.9466	92.85376	125.9184	74.48054
0.4	2.96313	3.47458	3.62908	5.44362	5.13229	2.04496	1.90164	3.64136	3.65811	123.3402	144.9471	92.85385	125.9181	74.48067

0.5	2.96384	3.47541	3.62995	5.44492	5.13352	2.04545	1.90209	3.64223	3.65899	123.3403	144.947	92.85385	125.918	74.4808
0.6	2.96455	3.47624	3.63081	5.44622	5.13474	2.04594	1.90255	3.6431	3.65986	123.3399	144.9468	92.85385	125.9178	74.48053
0.7	2.9649	3.47665	3.63124	5.44687	5.13536	2.04618	1.90277	3.64353	3.66029	123.3402	144.9475	92.85394	125.9186	74.48061
0.8	2.96561	3.47748	3.63211	5.44817	5.13658	2.04667	1.90323	3.6444	3.66117	123.3398	144.9473	92.85394	125.918	74.48066
0.9	2.96525	3.47707	3.63168	5.44752	5.13597	2.04643	1.903	3.64397	3.66073	123.3401	144.9467	92.85344	125.9181	74.48066
1	2.97374	3.48702	3.64207	5.4631	5.15066	2.05228	1.90844	3.65439	3.67121	123.3407	144.947	92.85398	125.9178	74.48083

Table S8. Variation of the interionic distances between the cations b, c, d, e, f, the cation-anion distance p, q, r, s, and the bond angles θ_1, θ_2, θ_3, θ_4, θ_5, of $Mg_{1-x}Co_xFe_2O_4$ nanoparticles annealed at 600^oC.

x	b	c	d	e	f	p	q	r	s	θ_1	θ_2	θ_3	θ_4	θ_5
0	2.96101	3.47209	3.62648	5.43972	5.12862	2.0435	1.90028	3.63875	3.65549	123.3397	144.9469	92.85366	125.9182	74.48069
0.1	2.96172	3.47292	3.62735	5.44102	5.12984	2.04399	1.90073	3.63962	3.65637	123.3399	144.9467	92.85366	125.9176	74.48081
0.2	2.96207	3.47334	3.62778	5.44167	5.13046	2.04423	1.90096	3.64006	3.6568	123.3402	144.9466	92.85376	125.9184	74.48054
0.3	2.96384	3.47541	3.62995	5.44492	5.13352	2.04545	1.90209	3.64223	3.65899	123.3403	144.947	92.85385	125.918	74.4808
0.4	2.9649	3.47665	3.63124	5.44687	5.13536	2.04618	1.90277	3.64353	3.66029	123.3402	144.9475	92.85394	125.9186	74.48061
0.5	2.96667	3.47872	3.63341	5.45011	5.13842	2.0474	1.90391	3.6457	3.66248	123.3398	144.9471	92.85403	125.9182	74.48079
0.6	2.96843	3.4808	3.63557	5.45336	5.14148	2.04862	1.90504	3.64788	3.66466	123.3404	144.9467	92.85371	125.9182	74.48046
0.7	2.96702	3.47914	3.63384	5.45076	5.13903	2.04765	1.90413	3.64614	3.66291	123.3401	144.9463	92.85353	125.9181	74.4806
0.8	2.97409	3.48743	3.6425	5.46375	5.15128	2.05253	1.90867	3.65483	3.67164	123.3399	144.9463	92.85349	125.9182	74.48057
0.9	2.97515	3.48867	3.6438	5.4657	5.15311	2.05326	1.90935	3.65613	3.67295	123.3398	144.9467	92.85358	125.9179	74.48069
1	2.97657	3.49033	3.64553	5.4683	5.15556	2.05424	1.91026	3.65787	3.6747	123.3396	144.9465	92.85358	125.9176	74.48055

Fourier-transform infrared spectroscopy (FTIR)

The force constants (F_C) for the A site (F_{CT}) and B site (F_{CO}) are obtained using the following relation

$$F_C=4\pi^2c^2\nu^2m \tag{30}$$

where ‘c’ is the speed of light~2.99×10^{10} cm s^{-1}; ‘ν’ is the vibration frequency of the A site and B site; ‘m’ is the reduced mass for the Fe^{2+} ions and the O^{2-} ions which is ~2.061×10^{-23} g[31]. Variations of F_{CT} and F_{CO} of $Mg_{1-x}Co_xFe_2O_4$ ferrites annealed at 200°C, 400°C, 600°C, and 800°C with Co^{2+} content, x are presented in Table S-9. The value of F_{CT} and F_{CO} increases with increasing Co^{2+} content x because the bond length of the A site and the B site decreases with an increase in Co^{2+} content x[41].

Table S9. Variations of F_{CT} and F_{CO} of $Mg_{1-x}Co_xFe_2O_4$ ferrites annealed at 200°C, 400°C, 600°C, and 800°C with Co^{2+} content, x.

	F_{CO}				F_{CT}			
x	200°C	400°C	600°C	800°C	200°C	400°C	600°C	800°C

0	160951.6	158649.5	156364.1	153342.6	356388.3	351819.2	342769.6	342769.6
0.1	158649.5	158649.5	154849.7	151843	352958.7	352958.7	340525.6	340525.6
0.2	159415	156364.1	156364.1	152591.9	350681.6	348411.8	341646.7	337173.5
0.3	156364.1	157124.1	153342.6	150350.7	348411.8	345020.9	337173.5	338289
0.4	157124.1	154849.7	151095.9	148865.7	342769.6	346149.4	334947.9	332729.7
0.5	155606	153342.6	151843	145185.6	345020.9	341646.7	336059.7	328315.4
0.6	154849.7	150350.7	149607.3	146652.1	340525.6	337173.5	332729.7	329416.2
0.7	152591.9	151095.9	147388.2	143726.5	341646.7	338289	329416.2	326119.3
0.8	153342.6	148865.7	145185.6	142274.7	337173.5	334947.9	327216.5	322839
0.9	151843	145918	143726.5	142999.7	338289	331623.4	322839	323930.6
1	149607.3	147388.2	144455.1	140830.3	334947.9	328315.4	323930.6	320661.4

Magnetization measurements

The values of saturation magnetization (M_s) were obtained by extrapolating M - $\frac{1}{H}$ curve at $\frac{1}{H} \rightarrow 0$ for a higher value of H[53]. The values of anisotropy constant (K) were obtained by using the relation,

$$K = \frac{H_c \times M_s}{0.96} \tag{31}$$

where H_c is the coercive field[31]. The variation of H_c, M_s, remanence ratio (M_r/M_s), and K with Co^{2+} content of $Mg_{1-x}Co_xFe_2O_2$ ferrite nanoparticles annealed at 200°C, 400°C, 600°C, and 800°C are presented in Table S10. The values of M_s, K, H_c, and M_r/M_s increase with an increase in Co^{2+} content and annealing temperature. The values of M_s increases with an increase in Co^{2+} content because the magnetic moment of Co^{2+} (3.88μ_B) is higher than that of Mg^{2+} (0μ_B). The values of H_c increases with an increase in Co^{2+} content because K increases with an increase in Co^{2+} content[51].

Table S10. The variation of H_c, M_s, remanence ratio (M_r/M_s), and K with Co^{2+} content of $Mg_{1-x}Co_xFe_2O_2$ ferrite nanoparticles annealed at 200°C, 400°C, 600°C, and 800°C.

Annealed at 200° C											
x	0	0.1	0.2	0.3	0.4	0.5	0.6	0.7	0.8	0.9	1
H_c	20	30	46	56	228	246	202	292	294	317	328
M_s	7.5	14.7	18.5	24.5	28.7	36.5	45.3	51.2	56.5	60.1	65.5
K	156.25	459.375	886.4583	1429.167	6816.25	9353.125	9531.875	15573.33	17303.13	19845.52	22379.17
M_r/M_s	0.01	0.01	0.01087	0.01071	0.0114	0.0187	0.02327	0.01644	0.03469	0.0489	0.04787
Annealed at 400° C											
x	0	0.1	0.2	0.3	0.4	0.5	0.6	0.7	0.8	0.9	1
H_c	15	20	30	55	167	200	220	276	278	381	411
M_s	13.5	18.1	20.9	27.9	32.6	38.5	47.9	53.4	58.2	63.6	70.8
K	210.9375	377.0833	653.125	1598.438	5671.042	8020.833	10977.08	15352.5	16853.75	25241.25	30311.25

M_r/M_s	0.015	0.016	0.02	0.025	0.02814	0.0275	0.03409	0.04601	0.0482	0.05328	0.05499
Annealed at 600º C											
x	0	0.1	0.2	0.3	0.4	0.5	0.6	0.7	0.8	0.9	1
H_c	30	50	80	150	265.4	326.5	382.9	486.6	643.3	702	784
M_s	14.5	21.2	24.2	29.1	34.5	42.4	50.2	55.8	63.6	72.9	85.5
K	453.125	1104.167	2016.667	4546.875	9537.813	14420.42	20022.48	28283.63	42618.63	53308.13	69825
M_r/M_s	0.02	0.023	0.025	0.0247	0.03145	0.03706	0.04623	0.04316	0.04731	0.05128	0.05102
Annealed at 800º C											
x	0	0.1	0.2	0.3	0.4	0.5	0.6	0.7	0.8	0.9	1
H_c	50	75	215.3	370.3	511.3	562.2	564	658.6	726.7	768	771
M_s	20	25.7	30.4	34.7	38.2	45.7	53.2	58.9	68.3	86.5	110
K	1041.667	2007.813	6817.833	13384.8	20345.48	26763.06	31255	40407.85	51701.68	69200	88343.75
M_r/M_s	0.04	0.043	0.046	0.047	0.04831	0.04909	0.05142	0.04995	0.05766	0.05469	0.08625

Hyperthermia Properties

Table S11 presents the variations of maximum attained temperature T_{max} with the particle size of chitosan-coated $Mg_{1-x}Co_xFe_2O_4$ nanoparticles having different concentrations while Table S12 presents the variations of specific loss power (SLP). The maximum attained temperature was determined from time dependence temperature curves in Figure 15. The highest temperature reached at 50 minutes was considered as T_{max} for each condition. The specific loss power was determined from the following relation,

$$SLP = \frac{c}{m}\frac{dT}{dt} \tag{32}$$

Where, C is the heat capacity of the solution, and m is the relative mass of the magnetic nanoparticles. The slope of the linear range of temperature vs. time curves provided dT/dt. The concentration of the magnetic nanoparticle is small, and therefore we considered the heat capacity of water to be 4.18 J g^{-1} K^{-1} for the sample.

Table S11. The variations of maximum attained temperature T_{max} with the particle size of chitosan-coated $Mg_{1-x}Co_xFe_2O_4$ nanoparticles having different concentrations.

	d (nm)	T_{max} for x=0.1	d (nm)	T_{max} for x=0.2	d (nm)	T_{max} for x=0.3	d (nm)	T_{max} for x=0.4	d (nm)	T_{max} for x=0.5	d (nm)	T_{max} for x=0.6	d (nm)	T_{max} for x=0.7	d (nm)	T_{max} for x=0.8	d (nm)	T_{max} for x=0.9	d (nm)	T_{max} for x=1.0
0.5 mg/ml																				
200ºC	4.9	38.9	5.5	39.6	5.6	41.5	6.2	42.9	6.8	45.6	7.6	46.4	7.9	47.9	8.1	53.5	8.9	58.8	9.6	78.5
400ºC	5.7	44.9	6.1	45	6.9	47.7	7.5	49.9	7.9	53.9	8.9	53.5	10.3	69.7	10.8	69	13.4	55	15.8	56.5
600ºC	11.5	49.9	12.5	53.9	15	53.5	15.6	47.4	15.9	45.2	16.5	40.2	18.6	45.5	21.8	39.8	23.6	34.9	24.9	32.7
800ºC	18.6	53.9	21.8	50.8	22.4	45.2	23.1	40.2	23.6	45.5	23.5	39.8	24.2	34.9	25.1	32.7	26.4	32.7	30.3	31.7
1.0 mg/ml																				
200ºC	4.9	40.6	5.5	42	5.6	42.9	6.2	46.4	6.8	48.2	7.6	49	7.9	49.8	8.1	56.5	8.9	67.1	9.6	84
400ºC	5.7	47.2	6.1	48.4	6.9	49.4	7.5	55.9	7.9	57.9	8.9	59	10.3	72	10.8	74	13.4	58.5	15.8	59.8
600ºC	11.5	55.9	12.5	57.9	15	59	15.6	56.3	15.9	47	16.5	41.5	18.6	46.2	21.8	43	23.6	36.4	24.9	35
800ºC	18.6	57.9	21.8	54.5	22.4	47	23.1	41.5	23.6	46.2	23.5	43	24.2	36.4	25.1	35	26.4	34.9	30.3	33
1.5 mg/ml																				
200ºC	4.9	42.6	5.5	44.1	5.6	44.9	6.2	47.9	6.8	51.8	7.6	52.4	7.9	53.8	8.1	60.8	8.9	75	9.6	90

400ºC	5.7	49.4	6.1	50.9	6.9	52.5	7.5	58.9	7.9	59.6	8.9	68.5	10.3	76.4	10.8	76.9	13.4	60.9	15.8	67.5
600ºC	11.5	58.9	12.5	59.6	15	68.5	15.6	64.8	15.9	51.4	16.5	47.8	18.6	49.2	21.8	45.2	23.6	39.5	24.9	37.3
800ºC	18.6	59.6	21.8	56.4	22.4	51.4	23.1	47.8	23.6	49.2	23.5	45.2	24.2	39.5	25.1	37.3	26.4	37.2	30.3	35.8
2.0 mg/ml																				
200ºC	4.9	46.5	5.5	46.9	5.6	48.2	6.2	52.8	6.8	56	7.6	59.4	7.9	63	8.1	68.9	8.9	80	9.6	94.5
400ºC	5.7	55.5	6.1	56.5	6.9	61.5	7.5	65	7.9	69	8.9	74.3	10.3	82.8	10.8	84.5	13.4	73.5	15.8	75
600ºC	11.5	65	12.5	69	15	74.3	15.6	68.3	15.9	60.6	16.5	55.9	18.6	53.7	21.8	50.6	23.6	46	24.9	43.6
800ºC	18.6	69	21.8	65	22.4	60.6	23.1	55.9	23.6	53.7	23.5	50.6	24.2	46	25.1	43.6	26.4	43.2	30.3	41.2

Table S12. The variations of specific loss power (SLP) with the particle size of chitosan-coated $Mg_{1-x}Co_xFe_2O_4$ nanoparticles having different concentrations.

	d (nm)	SLP for x=0	d (nm)	SLP for x=0.1	d (nm)	SLP for x=0.2	d (nm)	SLP for x=0.3	d (nm)	SLP for x=0.4	d (nm)	SLP for x=0.5	d (nm)	SLP for x=0.6	d (nm)	SLP for x=0.7	d (nm)	SLP for x=0.8	d (nm)	SLP for x=0.9	d (nm)	SLP for x=1.0
0.5 mg/ml																						
200ºC	4.3	122	4.9	165	5.5	169	5.6	183	6.2	211	6.8	266	7.6	291	7.9	286	8.1	353	8.9	438	9.6	792
400ºC	5.5	146	5.7	148	6.1	215	6.9	238	7.5	313	7.9	420	8.9	472	10.3	697	10.8	659	13.4	663	15.8	631
600ºC	10.1	238	11.5	313	12.5	420	15	472	15.6	270	15.9	276	16.5	283	18.6	277	21.8	251	23.6	148	24.9	153
800ºC	15.9	313	18.6	420	21.8	285	22.4	276	23.1	163	23.6	277	23.5	251	24.2	148	25.1	153	26.4	147	30.3	126
1.0 mg/ml																						
200ºC	4.3	71	4.9	91	5.5	94	5.6	99	6.2	107	6.8	266	7.6	176	7.9	160	8.1	209	8.9	321	9.6	398
400ºC	5.5	87	5.7	113	6.1	121	6.9	147	7.5	197	7.9	420	8.9	246	10.3	360	10.8	384	13.4	333	15.8	338
600ºC	10.1	147	11.5	197	12.5	248	15	246	15.6	177	15.9	276	16.5	157	18.6	167	21.8	106	23.6	76	24.9	80
800ºC	15.9	197	18.6	248	21.8	165	22.4	154	23.1	87	23.6	277	23.5	106	24.2	76	25.1	80	26.4	77	30.3	70
1.5 mg/ml																						
200ºC	4.3	55	4.9	52	5.5	55	5.6	55	6.2	74	6.8	93	7.6	95	7.9	98	8.1	110	8.9	191	9.6	214
400ºC	5.5	52	5.7	59	6.1	61	6.9	89	7.5	118	7.9	124	8.9	171	10.3	206	10.8	204	13.4	194	15.8	185
600ºC	10.1	89	11.5	118	12.5	124	15	171	15.6	120	15.9	94	16.5	101	18.6	88	21.8	67	23.6	45	24.9	48
800ºC	15.9	118	18.6	124	21.8	96	22.4	94	23.1	54	23.6	88	23.5	67	24.2	45	25.1	48	26.4	47	30.3	43
2.0 mg/ml																						
200ºC	4.3	34	4.9	39	5.5	47	5.6	47	6.2	47	6.8	50	7.6	58	7.9	82	8.1	108	8.9	119	9.6	123
400ºC	5.5	39	5.7	43	6.1	46	6.9	61	7.5	69	7.9	84	8.9	90	10.3	112	10.8	116	13.4	117	15.8	106
600ºC	10.1	61	11.5	69	12.5	84	15	90	15.6	71	15.9	66	16.5	59	18.6	48	21.8	41	23.6	42	24.9	39
800ºC	15.9	73	18.6	84	21.8	98	22.4	66	23.1	59	23.6	48	23.5	41	24.2	42	25.1	39	26.4	38	30.3	34
